# Synchronous Activity in the Auditory Forebrain Represents Temporal Landmarks in Vocal Communication Signals

**DOI:** 10.1523/JNEUROSCI.2108-25.2026

**Published:** 2026-09-29

**Authors:** Amirmasoud Ahmadi, Hermina Robotka, Manfred Gahr, Frederic Theunissen

**Affiliations:** ^1^Department of Behavioural Neurobiology, Max Planck Institute for Biological Intelligence, Seewiesen 82319, Bavaria, Germany; ^2^Graduate School of Systemic Neurosciences, Ludwig Maximilian University, Planegg 82152, Bayern, Germany; ^3^Department of Neuroscience, University of California, Berkeley, California 94720

**Keywords:** animal communication, auditory cortex, auditory objects, neural decoding, speech entrainment, zebra finches

## Abstract

Precise temporal segmentation of sound is critical for identifying and interpreting complex acoustic signals such as human speech (Pisoni and Luce, 1987) and bird song (Gentner, 2008). Using the zebra finch model system, we investigated the neural coding of the songs' temporal features. We recorded neural activity from avian primary and secondary auditory cortex-like brain areas using electrode arrays in awake birds (four males and three females) exposed to conspecific songs. By applying a deep-learning approach with bidirectional long short-term memory networks (Daly, 2023), we demonstrate that not only the amplitude envelope of song stimuli but also discrete, time-locked envelope landmarks can be decoded from multichannel neuronal responses to songs. The times of envelope peak amplitude and peak slope were decoded with average accuracies of 40% and maximum accuracies of 70% at 20 ms timescales. The decoding of these landmarks enabled the segmentation of song into syllables with maximum accuracy of 90% at 10 ms timescales. Decoding accuracy for temporal events was greater in the primary auditory than in the secondary auditory area, suggesting a serial processing of the segmentation and identification operation. These findings reveal that neural entrainment to salient temporal events in vocal communication signals is a ubiquitous, specialized feature of the vertebrate primary auditory forebrain (Mesgarani et al., 2009; Heelan et al., 2019) providing a mechanism for segmenting continuous acoustic streams into behaviorally relevant units for higher-order processing.

## Significance Statement

To decipher speech or vocal communication signals, humans and animals must segment continuous sound streams into identifiable syllables or call types. We found that amplitude envelope of songs and the time of salient acoustic landmarks can be decoded with high accuracy from the activity of neurons in the auditory cortex analog of songbirds. These landmarks can be used to segment the song into its component syllables. We found higher fidelity for the representation of the envelope of the sound in primary relative to secondary auditory regions. We postulate that this functional organization corresponds to the order of auditory computations where the segmentation task precedes the identification task.

## Introduction

Segmentation of dynamic acoustic input into tokens is a key computational task in the sound-to-meaning transformation (Pisoni and Luce, 1987; Gong et al., 2023). Once segmented, the sound units can be identified as morphemes, syllables, and words in human speech and as call types or specific song syllables in animal communication sounds. The neural mechanisms of auditory signal segmentation, however, remain unclear.

On the one hand, it is well established that auditory neurons in auditory cortical and cortex-like areas of vertebrates are tuned to the amplitude envelope of sounds (Osman et al., 2018; Penikis and Sanes, 2023). In both mammals and birds, distinct pallial auditory areas show distinct sensitivities to temporal rates (Sen et al., 2001; Woolley et al., 2009; Lim et al., 2016), allowing for the encoding of temporal patterns at different timescales. In humans, it is well known that neural signals recorded while subjects are listening to speech are entrained by the speech amplitude envelope (Ding and Simon, 2014). On the other hand, whether the amplitude envelope-based neural entrainment could be used to segment the sound stream remains unexplored. To segment the sound stream, one could determine relevant landmarks of the envelope. In human speech, the landmark theory (Stevens, 2002) postulates that specific acoustic and linguistic statistical features could be used for this purpose (Ding et al., 2016). Simple acoustic features include the peak of the sound's amplitude envelope and the peak of its derivative. It has been shown that, once such envelope landmarks are determined, they can be used to explain human neural responses to speech in auditory cortical areas: simple encoding models that replace each landmark by an impulse response weighted by an amplitude scale yield model response with high predictive power (Oganian and Chang, 2019). Here we ask whether the timing of such specific landmarks can be decoded directly from the neural response. Indeed, if segmentation is a key computational step in the sound-to-meaning transformation and if this segmentation requires the detection of specific landmarks, then a “landmark neural signal” should be present in the responses in auditory cortical areas. Specifically, we hypothesized that the song amplitude landmarks would be reflected in synchronized network activity and thus decodable either from the local field potentials (LFPs) or neural activity recorded simultaneously at multiple sites.

We tested this hypothesis in a songbird, the zebra finch. Zebra finches are a highly gregarious and vocal species, exhibiting a complex repertoire of calls and songs crucial for their intricate social communication (Gill et al., 2015; Elie and Theunissen, 2016). To enable specific vocal exchanges, these communication signals must be sequenced and identified. The zebra finch song, in particular, is a sequence of unique syllables (Sainburg et al., 2019) that need to be segmented to provide reliable information about the singer's identity or social status (Elie and Theunissen, 2018a; Geberzahn and Derégnaucourt, 2020; Loning et al., 2024). The avian auditory pallium (the analog of the mammalian auditory cortex) and its primary and secondary subregions have been very well characterized both in terms of basic response properties (Nagel and Doupe, 2008; Woolley et al., 2009; Lim et al., 2016; Spool et al., 2024) and its representation of song and communication calls in the zebra finch (Grace et al., 2003; Elie and Theunissen, 2015, 2018b; Robotka et al., 2023).

Using multielectrode neural responses obtained with electrode arrays targeted to record from primary and secondary auditory pallium, we assessed to what degree we could decode song events (syllables), the entire song amplitude envelope, and the time of two specific amplitude landmarks: peak amplitude and peak rate. To decode these features from neural data, we employed stacked bidirectional long short-term memory (BiLSTM) networks. BiLSTMs excel at learning long-term dependencies in sequential information, a crucial capability for processing neural signals related to complex acoustic sequences like speech or music (Anumanchipalli et al., 2019; Daly, 2023). We used the complex BiLSTM decoder to obtain an upper bound on the precision with which temporal landmarks could be decoded from neural responses. At the same time, by comparing the representations used by the artificial neural network in the activity of its hidden units to those found in our actual neural data, this decoding approach could yield insights on how the temporal information in represented and extracted in biological neural circuits.

## Materials and Methods

### Animals

We acquired the zebra finches (*Taeniopygia guttata*) from the Max Planck Institute breeding facilities. The four males and three females used in the experiments were 1–2 years old and weighed 19–21 g. During the recordings, the birds were housed in cages (120 × 50 × 50 cm) inside soundproof boxes. These boxes were equipped with microphones (Audio-Technica's USB microphone AT2020USB+) and speakers (FRS 8, 30 W, 8 U, VISATON) for audio recording and playback. In the sound box, zebra finches were kept at 24°C, 60–70% humidity, and a 14/10 light/dark cycle. To minimize electrical noise, the cage was enclosed within a custom Faraday cage. A transparent mesh divider was introduced in the middle of this cage during tethering, allowing pairs of implanted birds to maintain visual and vocal interaction without tangling their cables. When not recording, birds were released from their tethers and cohabited in the large cage without the mesh divider. All housing conditions were in accordance with EU regulations and experiments and surgical procedures were performed in compliance with national legislation on animal experimentation (animal testing license of the government of Upper Bavaria Az. 55.2-2532.Vet_02-19-131).

### Histology and reconstruction of the electrode tracts

At the end of the recording sessions, the positioning of the implanted array was verified after the chronic recordings were completed. We used a custom lesion device to perform electrolytic lesions on specific electrode channels and at multiple depths so that both the final depth and the electrode number could be identified. The output jumper cable was connected to the Omnetics connector on the array and a linear stimulus isolator (World Precision Instruments). To create the electrolytic lesions, 100 mA of current was applied for 5 s. These histological methods were also applied and described in Robotka et al. (2023).

The birds were killed with an overdose of isoflurane 30 min after electrolytic lesioning. The brains were carefully removed from their skulls. Dry ice was used to rapidly freeze the brains, which were then stored at −80°C until cryosectioning. Frozen brains were sectioned into 25 μm sagittal sections using a cryostat (Leica CM3050 S, Leica Biosystems). To localize the lesion sites and the electrode tracts, a light microscope (Leica DM 6000 B, Leica Biosystems) was used. Nissl-stained sections were photographed with a color digital camera (Leica DFC480, Leica Biosystems). We were able to identify the tracks of the electrodes in the rostral/caudal rows as shown in Figure 1 (see also Table S1). We were in this manner able to assign recording sites to the caudomedial nidopallium (NCM), caudomedial mesopallium (CMM), and the primary auditory nidopallium (Field L), as a function of depth and position along the rostral/caudal and medial–lateral position. Within Field L, we distinguished the L1 regions from the rest of the primary auditory nidopallium (L2a, L2b, L, and L3) based on differences in the density of neurons which is higher in L2a than in the dorsal neighboring region of the nidopallium. The depth of the recoding sites was estimated relative to the final recording position determined as the deepest point in our slices and the electrolytic lesion. There are however multiple uncertainties in this procedure. First, the depth assignment is approximative due to tissue shrinkage and the difficulty in assessing the “zero” point determined by the surface of the brain for the rostral electrodes. Second, we were not always able to visualize all 16 electrodes in our sections. To reduce these uncertainties and to fill in the gaps for the missing electrodes, we combined our histological observations with expected results obtained by “placing” the array in a published atlas of the zebra finch brain (http://www.zebrafinchatlas.org/gene_display/histological-atlas). For this step, the most caudal electrode penetrations in the histology were used to line up the rear of the array for each bird relative to the rostral/caudal position of the *y*-sinus. We also used a fixed 0.5 mm lateral value for the medial side of the array relative to the brain's midline since this was our standard coordinate for our penetrations. We then assigned locations to recording sites by combining the histological reconstructions obtained from each bird with the expectations obtained from the bird atlas (Table S1). Although this approach will give a coarse assignment into primary and secondary auditory regions within the pallium, it cannot assign recording sites to different regions of Field L: L2a, L2b, and L3. It will also lead to some miss-assignments for recording sites found close to the borders of L2 and L1, L1 and CMM, and Field L and NCM. To provide a complementary assessment of the relation between decoding accuracy and distinct auditory areas, we also report results based solely on recording depth.

**Figure 1.**
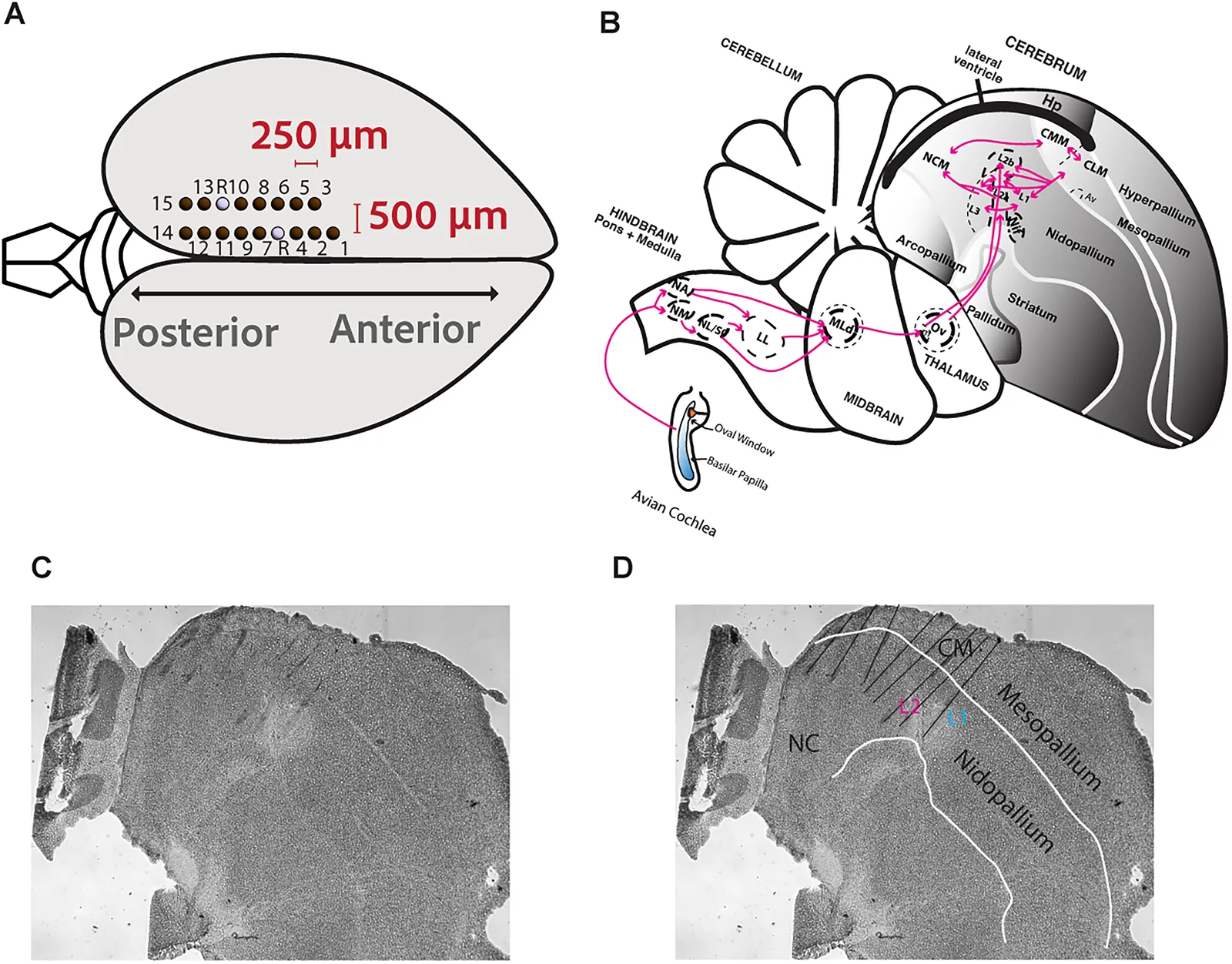
***A***, Neural signals were recorded from the left hemisphere auditory forebrain using 15-channel recording arrays with an interelectrode distance of 250 µm arranged in two rows 500 µm apart. Recording electrodes (filled circles) are numbered 1-15 and reference electrodes (empty circles) are labeled with “R.” The location of the electrodes and the recording sites within the brain were determined by combining data from histological reconstructions as in ***C*** and ***D*** with inferences obtained by placing the array in publicly available anatomical atlas (see Materials and Methods). Positions of recording sites used are shown in Table S1. ***B***, Diagram of the songbird ascending auditory pathway. Primary auditory pallial regions include the thalamo-recipient regions L2a and L2b and the lower and more superficial layers L3 and L1. Secondary auditory pallial regions include CMM and NCM. ***C***, Parasagittal Nissl-stained slice showing the final position for the lateral row (1 mm lateral from midsagittal sinus) of recording electrodes in the array for one subject bird. ***D***, The slice in ***C*** is annotated to delineate the lamina used to distinguish the mesopallium from the nidopallium and the nidopallium from the striatum. Density and size of stained neurons were used to approximate the boundary between L2a and the more superficial layer L1 as well as more rostral regions of the nidopallium.

### Surgery and electrode array implantation

Prior to the head-stage implantation, the birds received an injection of the painkiller metamizole and were deeply anesthetized with isoflurane. During anesthesia, they were maintained at a warm temperature using a heating pad and securely immobilized within a stereotaxic system. Next, preparations were made on the scalp and skull to create an entry point for electrode insertion. Subsequently, a custom-designed multielectrode array (platinum-iridium metal electrodes; shank diameter, 125 µm; tip diameter, 1–3 µm; 15 recording electrodes with impedance, 4 MW; two reference electrodes with impedances at 1 kW; MEA-PI/Ir from Microprobes for Life Science) was implanted into the avian auditory pallium (Fig. 1). The electrode array was affixed to an aluminum microdrive and positioned on the surface of the skull. A silver ground wire was carefully inserted, and the electrode tips were gradually lowered into the bird's brain. The craniotomy was sealed with DOWSIL 3-4680 (Dow Corning) acting as an artificial dura, and the microdrive was secured to the skull with dental cement (Tetric EvoFlow). To improve signal quality, adjustments were made to the array cable to minimize noise interference. Following the procedure, postoperative analgesia was administered to manage any discomfort, and the birds were allowed sufficient time to recover before being returned to their home cage, located within a soundproof box. For additional details, refer to Robotka et al. (2023).

### Neural data recording

Prior to the recording sessions, a male–female pair or zebra finches were placed in a cage within a soundproof box for 2 weeks for acclimatization and bonding. On the 10th day, the pair-bonded birds were equipped with backpacks attached to their bodies with rubber cords. To further help the birds acclimate to the recording setup, these backpacks were loaded with small dummy weights (∼1 g). A 16-channel amplifier board (RHD2132 16-Input Amplifier Board, Intan Technologies) connected to a head stage was used once the birds became comfortable with the weights. During recording, the amplifier in the backpack was connected to a computer with a slender cable (RHD2000 Ultra-Thin SPI Interface Cable; length, 0.9 m; Intan Technologies) via a slip ring commutator atop their cage (Dragonfly Research and Development). A grounding cable was routed outside the soundproof enclosure and connected to the ground input on the interface board to ensure proper electrical grounding. A 25 kHz amplifier sampling rate was used to record neural responses from the 15-channel multielectrode array signal. Gradually, electrodes were lowered into the brain until auditory neurons were discernible. We then advanced the electrodes every day by 0.5–1.0 turns (one turn equals 250 µm) in the dorsoventral direction and recorded. The recording depths are between 125 and 2,635 µm (mean ± SE, 1,420 ± 12 µm). We collected data for 20–30 d until the electrode tips reached depths of between 2.25 and 2.75 mm.

The slip ring commutator allowed the birds to freely move within their cage. Since the birds' bodies generated the torque on the commutators, this setup also increased the birds' comfort and mobility. In these amplifier boards, neural signals recorded from 15 electrodes were filtered, amplified, and digitized. The birds were tethered near their center of mass, allowing them to maintain mobility and comfort. During the recording sessions, the bird pairs were separated within their cage by a plastic wall, consisting of a frame and mesh, which allowed them to maintain visual and auditory contact while being tethered. With this setup, birds engaged in typical behaviors, including vocal communication. Despite continuous neural recording being possible, experiments were limited to 4–8 h during the day.

We presented sound stimuli in a pseudorandom order with an interstimulus interval ranging from 3 to 6 s using custom Python code from the Gentner lab (https://github.com/gentnerlab/pyoperant). At each recording site, we played 10 distinct songs, all of which were unfamiliar to the birds. Each song was played 10 times to ensure a comprehensive data collection. The output to the speaker and the USB microphone signal were connected to the analog inputs of the Intan RHD USB interface board and, in this manner, were synchronized with the neural data and stored in a single file on the computer. Song playbacks were selected by experts who only considered recordings of clear quality, defined as a signal-to-noise ratio >20 dB. To avoid cognitive and attentional effects, all songs were unfamiliar to the birds; while the neurophysiological recordings were performed at the MPI of Biological Intelligence in Seewiesen, the song stimuli were recorded at UC Berkeley. All songs exhibited the typical zebra finch song structure, including introductory notes and motifs. The selected songs varied in syllable complexity and temporal features, with durations between 1.7 and 2.5 s. The spectrogram of an example playback song is shown in Figure 2*D*. All raw electrophysiology data with playbacks wave files are available in https://doi.org/10.17617/3.UINR2V.

### Neural data preprocessing and feature extraction

Neural signals were recorded from seven birds across 51 sites corresponding to different recording depths or the same depth but recorded on different days. Neural signals were synchronized with playback times, and acoustic data were simultaneously recorded using two microphones. We segmented the neural data into epochs spanning from 1 s before playback onset to 1 s after playback offset. To ensure that the playback stimuli were delivered as intended, we validated the quality of the acoustic data captured by the microphones. Since our primary focus was on analyzing responses to playback song stimuli, we excluded trials that followed live vocal communication between two birds; these accounted for >2% of the total trials. To mitigate the noise introduced by the birds' unrestricted movements and flight, which could affect certain trials, we identified and excluded trials exhibiting excessive noise in the LFP signals. After preprocessing with notch filtering at 50 Hz and a 1 Hz high-pass filter, specific statistical features, including signal variance, kurtosis, maximum amplitude, and maximum voltage gradient, were extracted for each trial. Trials exhibiting metrics outside the mean ± 3 SD obtained for the entire session were classified as artifacts. In addition, any trial exhibiting an absolute amplitude exceeding 400 µV across more than half of the electrode array (≥8 channels) was excluded from subsequent analyses. After all preprocessing steps, we retained 71.76 ± 0.44% (mean ± standard error) of the initially recorded trials. From these clean neural signals, we extracted LFPs, multiunit activity envelope (MUAe), multiunit spikes (MUA), and single-unit spikes.

#### LFPs

In Figure 2*B*, we illustrate key additional steps used for processing the neural signal to extract LFPs. We applied a second-order Butterworth bandpass filter (1–200 Hz) to the neural signals to extract LFPs. The LFPs were then downsampled to 1 kHz. To enhance signal quality, denoising is performed using a common average reference (CAR) filter. In this approach, noise is assumed to be a component common to all channels that can be effectively removed by subtracting the mean of all channels from each channel. Afterward, the LFP signals are further processed through Butterworth filters to segment them into common frequency bands: delta (1–4 Hz), theta (4-8 Hz), alpha (8-13 Hz), low beta (13-20 Hz), high beta (20-30 Hz), low gamma (30-50 Hz), mid gamma (50-100 Hz), and high gamma (100-150 Hz). To smooth the filtered signals, a third-order Savitzky–Golay filter with a window length of 10 ms was applied. These smooth narrow-band signals were all sampled at 1 kHz, well above twice the Nyquist limit given by the upper frequency (300 Hz).

#### MUAe

The MUAe was extracted by the following steps (Ahmadi et al., 2021): after filtering the neural signals between 500 and 9,000 Hz, the signals were rectified, followed by additional filtering in the range of 1-200 Hz and then downsampled to 1 kHz. The 200 Hz upper limit of the smoothing filter is above the frequency of coherent time spiking across trials (Hsu et al., 2004). The sampling frequency matched the one used for the LFP.

#### MUA

Multiunit activity (MUA) was obtained by thresholding the 500-9 kHz neural signal to detect spike events. These spike snippets were not sorted and thus classified as multiunits.

#### Single units

Single units were found by spike sorting using the Kilosort 2.5 toolbox (Pachitariu et al., 2016), an advanced computational method designed for high-density electrophysiological data: it applies a drift correction algorithm to account for electrode displacement and signal variability over time, ensuring stable spike detection across the entire recording duration. Kilosort 2.5's automated approach allowed for high-throughput, reliable spike sorting across multiple channels, yielding a final set of units with consistent spike waveforms and low refractory period violation [see also Robotka et al. (2023) for additional quantification of the quality of single-unit isolation]. A total of 423 single units were sorted from different brain sites and regions.

### Data analysis

We applied preprocessing steps to reduce data noise and segmented the continuous signals into individual playback trials. Extracting neural features from these segmented signals was a crucial step, as visually represented in Figures 1*B* and 2*A*. We analyzed these neural signals to automatically decode four temporal features of their songs based on amplitude envelopes (see below). The extracted features were then used to train a deep neural network (DNN) to decode these song features, with the architecture illustrated in Figure 2*C*. This network was specifically designed to decode four distinct temporal song features, as depicted in Figures 2*D* and 3*B*. The temporal acoustic features we examined are song events, the entire amplitude envelope, and two amplitude envelope landmarks, being the peak of the amplitude envelope (PeakEnv) and the peak of its derivative (PeakRate).

#### Song events

These are the beginning and end of sound chunks that compose the song. We used the term “song event” instead of “song syllables,” because song syllables are often manually extracted from visual inspection of the spectrogram and our events might not correspond to what a birdsong researcher could refer to as the song syllable, although we believe that any discrepancy will be small. The song events were detected based on the root mean square (RMS) envelope of the song waveform bandpass filtered between 100 and 5,000 Hz. The RMS envelope estimates the envelope of a signal by calculating the RMS value of the signal's amplitude within a short sliding time window. As the window slides across the entire signal, the sequence of these RMS values forms a new signal that traces the overall amplitude trend or “envelope” of the original signal, effectively smoothing out the rapid oscillations and highlighting the slower, broader changes in loudness or intensity. The formula of the RMS is as follows:

$$\mathrm{En}v_{\mathrm{RMS}}(k)=\;\sqrt{\frac{1}{\mathrm{WL}}\sum_{n=-WL/2}^{WL/2}\mathrm{Am}p_{\mathrm{song}}{\left(k+n\right)}^{2}\;}.$$

The *k*th sample of the RMS envelope, denoted as some variable (Env_RMS_), is calculated using a specific formula (Formula 1). In this formula, the window length is WL = 500 samples, which corresponds to a total window duration of 20 ms. After computing the RMS envelope, its sample rate is reduced from 25,000 to 1,000 Hz. This downsampling step is performed to match the frequency characteristics of the neural signals being analyzed.

To identify song events, we used an interval starting 200 ms before the playback sound onset to 200 ms after the offset. Time intervals during which the RMS envelope exceeded a threshold value were labeled as syllable events, while intervals during which the RMS envelope remained below were classified as silent segments. The threshold value was set at one standard deviation of the song amplitude envelope. For our decoding analysis, the syllable events were sampled at 100 Hz: in each 10-ms time window, an event feature is set at either zero or one, where zero denotes the silent intervals, and one indicates the syllabic segments (Fig. 3*B*). When we describe the decoder performance and state, for example, that a song stimulus of 2,500 ms or 250-time windows is decoded with 90% accuracy, we mean that we estimated correctly the value of sound versus silence in 235/250 time windows.

#### Amplitude envelope

Figures 2*D* and 3*B* provide a visual representation of the amplitude envelopes associated with our song playbacks. We used the narrow-band envelope derived using the loudness method (Schotola, 1984). This particular envelope type exhibits higher-frequency temporal changes and more rapid variations than the RMS window defined above, making it especially adept at revealing intricate details within song playback envelopes. These amplitude envelopes are computed and decoded at a 2 ms sampling rate, offering a precise representation of a song's temporal characteristics. This method extracts the analytic envelope of the song signal filtered within critical bands based on the Bark scale (Zwicker and Terhardt, 1980). This is achieved by square-rectifying the signal within each filter bank, averaging across all bands, and bandpass filtering between 1 and 10 Hz.

#### Envelope landmarks

We used the peak of the amplitude envelope (PeakEnv) and the peak of its derivative (PeakRate) as landmarks (Fig. 3*B*). These were coded as one-hot time series sampled at 20 ms.

### BiLSTM

We used a stacked BiLSTM network to decode distinct temporal features from neural signals (Fig. 2*C*). Stacked BiLSTM is a recurrent neural network designed specifically for sequential data processing (Daly, 2023). The network architecture we used consisted of two interconnected BiLSTM layers, with dropout layers (Baldi and Sadowski, 2013) strategically inserted after each layer to mitigate overfitting during training. For song event and amplitude envelope landmark classifications, the second BiLSTM layer is fully connected to the output layer that is read out with a SoftMax function, yielding the target class count. We optimized the network using the Adam optimizer (Zhang, 2019), with training constrained to a maximum of 50 epochs and a minibatch size of five. Network hyperparameters, including neuron count, initial learning rate, and dropout rates, were systematically optimized through Bayesian optimization. By evaluating performance across randomly selected recording sites, we identified optimal configurations: 200 neurons, a 0.5 dropout rate, and an initial learning rate of 0.01.

For amplitude envelope decoding tasks, we augmented the fully connected and SoftMax output layers with a rectified linear unit activation function (Banerjee et al., 2019). These specific tasks employed an initial learning rate of 0.001 and a minibatch size of 10, with configurations closely mirroring those of the sequence classification tasks.

Before performing the decoding analysis, we matched the sampling rate of our stimulus features and neural signals. Here, the neural signals are the LFP decomposed into the common frequency bands (see above) and the MUAe. For decoding song events, the neural signals were filtered between 1 and 50 Hz; for decoding the amplitude envelope, between 1 and 150 Hz; and for decoding the envelope landmarks, between 1 and 25 Hz.

### Quantifying the decoder performance

To provide a comprehensive assessment of the performance of the decoder, we used four performance metrics: classification accuracy, Cohen's kappa coefficient, correlation coefficient, and coefficient of determination. To ensure generalization, these measures of goodness-of-fit were estimated using two different variants of cross-validation (CV): the leave-one-out CV (LOOCV), and 10-fold CV.

For LOOCV, we treated each trial as an individual recording session containing song data. The LOOCV was used to assess the decoder performance for song events and envelope landmarks. For the 10-fold CV, the entire dataset was randomly partitioned into 10 equal-sized subsets or “folds.” In each of the 10 iterations, one fold served as the test set, while the remaining nine folds were combined to form the training set. The model was trained and then tested on the test fold in each iteration. After completing all iterations, we averaged the performance metrics across all folds to obtain a comprehensive assessment. In contrast to LOOCV, this approach uses larger samples for testing the dataset for each trained network, which can provide a more reliable evaluation, especially useful for smaller datasets. It is also computationally more efficient. We used the 10-fold CV to assess the decoder performance for the complete amplitude envelope.

To quantify the decoder's performance for the song event and envelope landmark detection, we used the classification accuracy and Cohen's kappa coefficient. Classification accuracy is defined as the proportion of time samples the model correctly classified. It is calculated by dividing the number of correct predictions by the total number of predictions. Note that while accuracy is a straightforward metric suitable for balanced datasets where each class has a similar number of samples, it can be misleading in the case of imbalanced datasets. For instance, if 90% of the data belongs to one class, a model could achieve 90% accuracy by always predicting that class, even if it fails to detect the minority class effectively. Therefore, in the text, we compare the accuracy of the decoder to the null model based on average class distribution and using permutation tests for which the song event vector was randomized. Cohen's kappa coefficient is a statistic that measures the agreement between the model's predictions and the actual labels, adjusted for chance agreement (Kuhn and Johnson, 2013). Kappa is calculated using the following formula (Vieira et al., 2010):

$$\mathrm{Kappa}=\;\frac{\mathrm{Ac}c_{\mathrm{Obs}}-\;\mathrm{Ac}c_{\mathrm{expect}}}{1-\;\mathrm{Ac}c_{\mathrm{expect}}}.$$

In the formula, $\mathrm{Ac}c_{\mathrm{Obs}}\;$ is the average of the observed per-class accuracy, and $\mathrm{Ac}c_{\mathrm{expect}}\;$ is the expected average accuracy by chance, calculated based on the average class distribution.

To evaluate the neural network performance on the envelope decoding task, we used two measures of goodness of fit: the Pearson’s correlation coefficient (*r*) and the coefficient of determination (*R*^2^) between the predicted envelope and the actual envelope of the song.

To benchmark the BiLSTM decoder, we compared its envelope decoding performance against the linear multivariate temporal response function (mTRF; Crosse et al., 2016). The mTRF decoder was trained using a temporal integration window of 50 ms and an L2 regularization parameter selected from a logarithmic grid ranging from 5^−^^6^ to 5^6^ via CV. The linear mTRF was evaluated on identical training, validation, and test partitions and using the same 10-fold CV scheme as the BiLSTM, with performance quantified by the Pearson’s correlation coefficient (*r*) and the coefficient of determination (*R*^2^) between the predicted and actual song envelope. The results for that comparison are shown in Figure S1.

### Recording electrodes contribution

To evaluate individual electrode contributions to a neural network's decoding performance, we used a permutation analysis. The process begins by first training the neural decoder using the unaltered, original data from all electrodes. This initial model training establishes a baseline measure of performance, often represented by a decoding accuracy score on an unmodified test set. This baseline score, which we will refer to as *D*_All_, reflects how accurately the model can decode with all 15 electrodes.

With this baseline established, we then systematically assess the contribution of each electrode by permuting or shuffling only that electrode's data in the test set while keeping the data from all other electrodes unchanged. For each electrode, we shuffle its values randomly across trials in the test dataset and then measure the model's performance on this altered dataset. This new performance score, labeled $D_{\mathrm{perm}}^{\left(i\right)}$, captures how well the model decodes when the information from the specific electrode “(1)” is eliminated. The impact of each electrode is then quantified by calculating the difference between the baseline performance and the permuted performance for that electrode, represented as $\Delta D(i)=\;D_{\mathrm{All}}-\;D_{\mathrm{perm}}^{\left(i\right)}$. If the shuffling of an electrode's data leads to a large drop in decoding accuracy, it indicates that this electrode is highly important for decoding, as its unique signal pattern contributes meaningfully to the model's performance. Conversely, if permuting the data from an electrode has a minimal effect on decoding accuracy, it suggests that the electrode's information may be less crucial to the model's predictive success.

Furthermore, to generate a metric that quantifies a single electrode decoding performance, we reasoned that this statistic should combine both the overall performance obtained with all the electrodes and the contribution of a particular electrode. Thus, we defined an effective decoding index (EDI) as follows:

$$\mathrm{EDI}(i)=D_{\mathrm{All}}\cdot \left(1+\frac{\Delta D(i)}{\Delta D(i_{\max})}\right)/2,$$

where $\Delta D(i_{\max})$ is the maximum decrease in performance obtained when one shuffles the *i*_max_ electrode. The EDI is therefore the decoding that one obtains by averaging the overall decoding performance with a value obtained by removing the normalized contribution of a single electrode. When ΔD(i)=0 (the electrode has little effect on the decoding performance), the EDI is set to half of the decoding performance. This is the smallest value that the EDI can take. Note that ΔD(i)=0 does not mean that the electrode is not informative but that, instead, its contribution to the decoding is redundant with that of the other 14 electrodes. When $\Delta D(i)=\Delta D(i_{\max})$ (the electrode is the most uniquely informative in the array), the EDI is equal to the decoding performance obtained with all the electrodes.

### Single-unit analyses

From the 423 single-unit data, we performed multiple additional analyses. We used the Gaussian copula approximation to estimate the mutual information (MI) between the stimulus at time *t* and the neural response, also at time *t* (Ince et al., 2017; code: https://github.com/robince/gcmi). This measure of instantaneous MI is a lower bound on the actual instantaneous MI that one could estimate by finding the optimal delay and/or fully characterizing the joint probability distributions. On the other hand, note also that the instantaneous MI cannot be used (e.g., simply by multiplying by the sampling rate in hertz) to obtain the actual MI between the two time series. By not taking into account the correlations, one risks a gross overestimation of the actual MI (Elie and Theunissen, 2019). The instantaneous MI calculated here is akin to a correlation coefficient, but it takes into account nonlinear relationships (De Clercq et al., 2023).

We estimated the instantaneous MI in two contexts: first, between the neuronal firing rate and discrete song events (i.e., the vector of (0,1) values specifying occurrence of song syllables) and, second, between the firing rate and the continuous envelope of the song, representing temporal fluctuations in song amplitude. This allowed us to calculate the information rate of neurons in both song event and envelope decoding scenarios. In the song event scenario, single-unit firing rates [*f*_event_(*t*)] were calculated by counting spikes in 50 ms bins shifted every 10 ms. These firing rates were then normalized using the baseline firing rate ( $\underline{\;f_{0}}$), defined as the mean firing rate during the 500 ms period preceding stimulus onset:

$$f_{\mathrm{Normal}\;}(t)=\;\frac{\;f_{\mathrm{event}}(t)-\underline{\;f_{0}}\;}{\underline{\;f_{0}}}.$$

To identify single units that track song envelopes, we calculated neuronal firing rates by counting spikes in nonoverlapping 2 ms bins. The song envelopes were also computed using 2 ms time windows, as detailed above (De Clercq et al., 2023). For completeness, we also evaluated the cross-correlation between the firing rate of neurons in nonoverlapping 10 ms bins and the timing of envelope landmarks, PeakRate, and PeakEnv. As explained above, this correlation measure captures the linear fraction of the stimulus-response relationship.

### Intertrial phase coherence (ITPC)

The phase coherence of oscillations quantifies the degree of temporal synchronization while allowing for systematic frequency-dependent delays between the two time series (Lachaux et al., 1999). In neurophysiological experiments based on multiple trials, the ITPC provides a rigorous quantification of the phase synchrony (Cohen, 2014). ITPC can be computed across various frequency bands and thus can measure phase synchrony both within and across frequency bands. For each experimental session and stimulus (song), we generated a two-dimensional time–frequency map (Fig. S6*A*). These maps display time points along the horizontal axis and frequency bands along the vertical axis, with *z*-scored ITPC (ITPCz) values represented by color intensity.

For each frequency and time point, we calculated the phase angle of the complex-valued wavelet coefficients. The index of ITPC was determined by summing the phase angles of all epochs using the following formula:

$$\mathrm{ITPC}=\;\|\frac{1}{N}\sum_{n=1}^{N}e^{i\emptyset_{n}}\|.$$

*N* represents the total number of trials and recording channels within each recording session and song, and $\emptyset_{n}$ is the phase angle of the wavelet coefficient in radians. A value of one indicates perfect phase coherence, while a value of zero indicates a random phase distribution across trials and recording channels. As a result of variations in the number of trials between conditions, we standardized the ITPC values to be $ITPCz=n*ITPC^{2}$. Using Rayleigh's *Z* test, ITPCz is approximated as statistically significant when >3 (*p* = 0.05; Cohen, 2014).

Our dataset comprised 51 recording sites spanning 10 unique songs. For each song and session combination, we computed the mean ITPCz across the song's duration and measured the corresponding decoding performance. For each song, we extracted 51 paired values (one from each session), consisting of mean ITPCz values for specific frequency bands and their corresponding decoding performance scores. We then calculated correlation coefficients between these paired measurements. The resulting analysis yielded 10 correlation values per frequency band (one for each song), which we plotted as individual points. This visualization reveals the strength of the relationship between phase coherence and decoding accuracy across different frequency ranges. Results for one song and three recording sites are shown in Figure S6, *A* and *B*.

### Neural network latent space

We investigated the latent representations of our DNN to gain insight into the mechanisms of temporal feature decoding (Fig. 8). We calculated the activities of the hidden neurons (artificial neurons) in each layer to examine how decoding occurs from the input features. Principal component analysis (PCA) was used to project the neural activity of hidden neurons during song playback onto one dimension, allowing us to visualize the similarity between the hidden neuron activities in each layer and the target amplitude envelope of the songs. The correlation coefficients between the actual envelope and the hidden neurons' activity projected onto the first PC are displayed at the top of each plot. To visualize and analyze (cluster) the artificial neurons' activity patterns, we used the nonlinear embedding method called the *t* distributed Stochastic Neighbor Embedding (*t*-SNE).

Our analysis pipeline consisted of three main steps. First, in the data preprocessing phase, we computed single-unit firing rates using 2 ms temporal bins, followed by Gaussian smoothing with 100 ms windows to reduce noise while preserving low-frequency temporal dynamics. Next, for dimensionality reduction, we applied *t*-SNE with optimization by the Barnes–Hut algorithm. The algorithm utilized a *k*-nearest neighbors search with the Euclidean distance metric and 30 local neighbors per point (perplexity), chosen to balance local and global structure preservation. We set the learning rate to 500 to ensure convergence while avoiding local minima and implemented a maximum iteration count of 1,000 with early stopping if convergence was achieved. To ensure reliability, we validated the stability of *t*-SNE embeddings across multiple random initializations. Third, in the clustering analysis phase, we employed DBSCAN (density-based spatial clustering of applications with noise) for unsupervised clustering. We optimized the clustering parameters through grid search, selecting an epsilon (neighborhood radius) of two and a minimum number of neighbors of six. The distance metric used in DBSCAN was the squared Euclidean distance, enhancing the method's sensitivity to differences in the high-dimensional space and improving cluster detection. The latent-space analysis shown in Figure 8 was performed on the envelope decoder trained on multichannel LFP features. For the comparison with single units (Fig. 9), the artificial neurons were the hidden units of the second BiLSTM layer of the envelope decoder trained on multichannel MUAe from a single recording session (Session 46). The single units were pooled across all birds and recording sessions; 29 of the 423 units (6.9%) were recorded in Session 46 and thus contributed indirectly to the input of this decoder.

To compare actual (biological) and artificial neuronal patterns, we visualized the single-unit activities alongside the artificial neurons' activities (Fig. 9). To assess whether this comparison depended on units that contributed to the decoder input, the joint embedding and clustering were repeated across 10 random *t*-SNE initializations, with and without the 29 single units recorded in session 46. Mixed clusters were defined as clusters containing at least five artificial and five actual neurons, with artificial neurons forming 20–80% of members. Overrepresentation of Session 46 units in each mixed cluster was tested with a one-sided Fisher's exact test, and the similarity between artificial and actual neurons in each mixed cluster was quantified as the Pearson’s correlation between their mean activity profiles. Furthermore, we verified that the identified clusters were not artifacts of the dimensionality reduction process and assessed the preservation of distances between the high-dimensional and low-dimensional representations.

### Statistical analysis

Statistical analyses were central to assessing decoding performance, comparing neural signals, and understanding the underlying neural mechanisms. The use of linear mixed-effect models was essential due to the nested structure of our data (trials within sites, sites within birds). These models let us consider the impact of specific “fixed effects” (e.g., neural signal type (LFP vs MUAe), recording depth, song stimulus identity, specific LFP frequency bands, or song segment) on decoding accuracy while accounting for variability across individual birds and recording sites, which were included as random effects. Model comparisons, using likelihood ratio tests (reported as *χ*² statistics), were used to determine whether including a specific fixed effect significantly improved the statistical model's fit, indicating a significant impact of that factor on the performance of our DNN decoder.

Regional differences were also modeled with linear mixed models (lme4, R 4.4.2) specifying brain region, sex, and recording depth (*z*-scored) as fixed effects and nested random intercepts [1 | Bird/Site] (Type III *F* tests, Satterthwaite df; lmerTest); estimated marginal means (EMMs) and Tukey-adjusted pairwise contrasts were obtained via emmeans.

ITPCzs were computed for LFP signals to measure the consistency of neural phase responses across repeated trials within specific frequency bands (alpha, beta, gamma, delta, theta), which gives a measure of neural entrainment to the stimulus. The significance of the correlation between the strength of this phase coherence (mean ITPCz per band) and the accuracy of decoding temporal song features (events, envelope, landmarks) was also explicitly assessed using linear mixed-effect models, again controlling for bird and song number as random effects.

## Results

Neural signals were recorded from primary (Field L separated into L1 and the other Field L regions) and secondary (NCM, CMM) auditory pallial regions using electrode arrays with 15 recording electrodes in response to conspecific songs in awake, freely behaving zebra finches (*n* = 7: 4 males, 3 females) during passive playback listening (Fig. 2*A*). The neural signals were processed to extract LFPs, MUA based on the rectified envelope of the high-pass filtered neural signals (MUAe), MUA composed of the times of unsorted spike snippets from thresholded high-pass neural signal, and single units obtained by spike sorting these snippets. In each bird, we recorded neural signals at various dorsoventral depths of the pallium by mechanically moving the electrode array. We obtained a total for 51 recording sites across all birds and across all 51 recording sites, the responses of 432 single units (see Materials and Methods).

**Figure 2.**
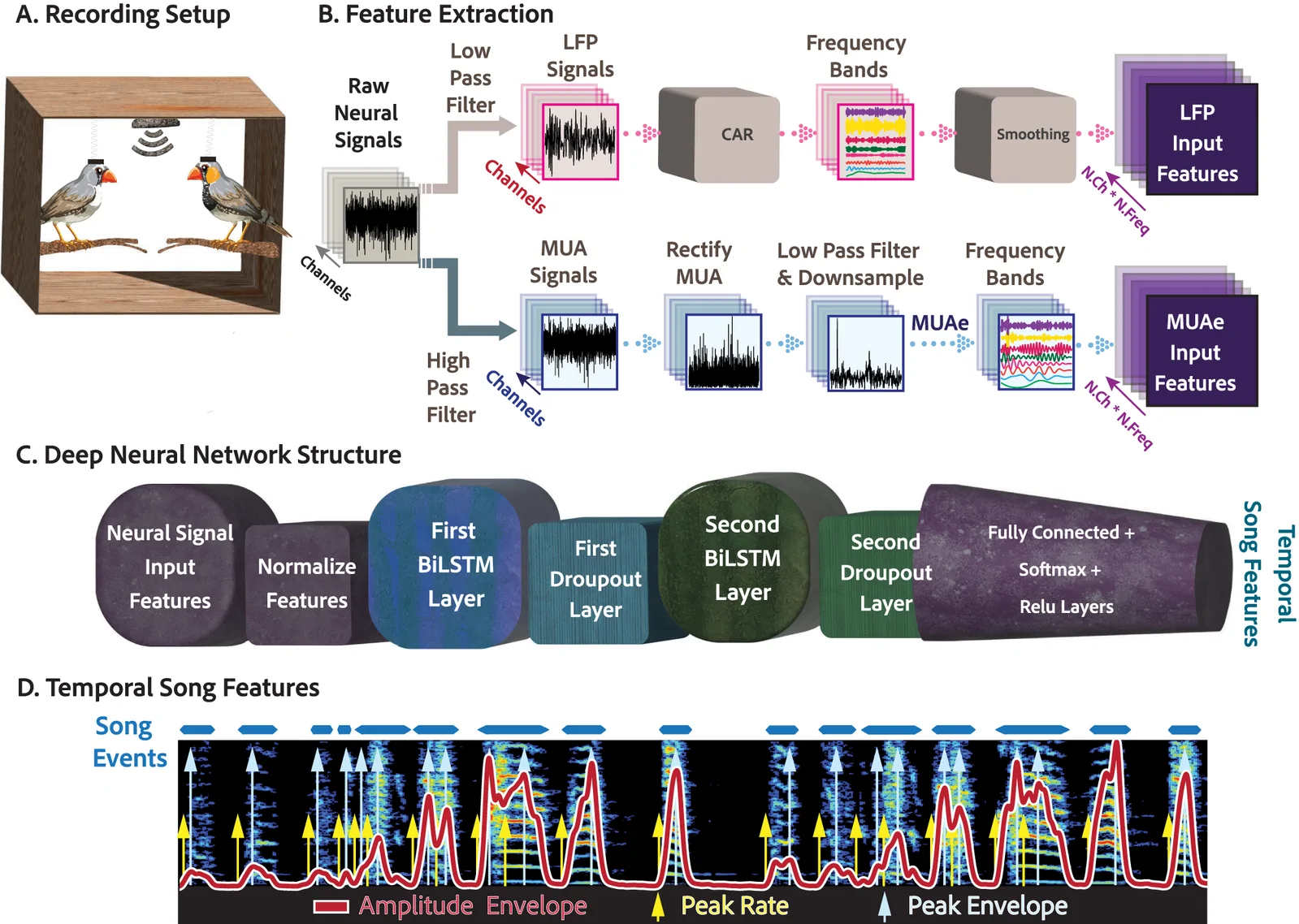
Neural-based decoding of zebra finch song temporal features. ***A***, Experimental setup and recording array. Neural data in response to song playbacks were obtained in freely behaving but tethered birds. Mated birds were housed and recorded together and could interact visually and acoustical via a mesh partition when tethered. ***B***, Steps used for extracting LFP and MUA (MUAe) features from raw neural signals. CAR, common average reference. ***C***, DNN architecture used for decoding song temporal song features. BiLSTM, bidirectional long short-term memory. ***D***, Decoded temporal song features. The temporal song features consist of song events (sound vs silence, shown in blue), the amplitude envelope (red line), and the PeakRate (time points of maximum positive slope, indicated by yellow arrows), all within the same PeakEnv (marked by light blue arrows). The panel displays a spectrogram of a song stimulus and the four decoded temporal features of an example song stimulus.

We decoded temporal features of zebra finch songs from the LFPs and MUA (MUAe). For both the LFP and the MUAe, the decoder was based on the multichannel response obtained using the 15 signals simultaneously but using single trials set. Trials in the training set were distinct from those in the testing set (see Materials and Methods, Quantifying the decoder performance). We wanted to estimate this single-trial decoding performance because we postulated that the neural representation of temporal events would be found in the synchronous activity across multiple neurons; thus, we only used multichannel LFP and MUAe as inputs to the decoder. The MUA and single-unit data were then analyzed to relate the neural activity found at particular electrodes with the overall decoding accuracy estimated from all electrodes. Additional preprocessing steps of LFP and MUAe extraction are schematically presented in Figure 2*B*. The decoder was a stacked BiLSTM DNN (Fig. 2*C*; see Materials and Methods for details).

The temporal events that were decoded included the song syllables, called song events from here on, and two amplitude envelope landmarks, the peak of the amplitude envelope (PeakEnv) and the peak of its derivative (PeakRate). The song events were determined by thresholding the amplitude envelope. We use the term “song events” and not “song syllables” because these units might not always correspond to song syllables as defined traditionally in the field. Finally, to obtain an overall assessment of temporal fidelity agnostic to the choice of temporal events, we also assessed how well we could decode the complete time-varying amplitude envelope.

### Neural decoding of temporal features

For each recording site (*n* = 51), we trained the DNN decoder using the multichannel LFPs and MUAe signals, respectively, to reconstruct the temporal features of the stimulus. A recording site is defined by the specific depth of the electrode array, where depth 0 is the entry point into the brain by the most rostral and caudal electrode in the array. As an example, Figure 3 shows the result of our decoding analysis for one site, one song stimulus, and one particular trial. The LFP obtained in 6 of the 15 electrodes was decomposed into eight frequency bands commonly used in LFP analyses (delta to high gamma; see Materials and Methods; Fig. 3*A*). This frequency decomposition yields a set of narrow-band signals that can be more easily further processed to extract the information bearing features present in the LFP signal. The DNN decoding allowed us then to assess whether information bearing features about the temporal landmarks were present in the neural response and to what degree. The results are striking because all the temporal landmarks of the stimulus we tested could be recovered with high precision (Fig. 3*B*). In the song event detection (Fig. 3*B*, top row), the decoder accurately identifies two distinct states, the events and silences. With an accuracy exceeding 90% (calculated as the percentage of correct predictions in 10 ms windows out of total song time samples), the decoder effectively captures the onset and offset of songs, as well as individual song events. The dynamics of the song envelope could also be decoded accurately (cross-validated Pearson’s *r* = 0.77, *R*^2^ = 0.6, for this song and trial, *p* < 10^−4^, permutation test). Finally, we were able to decode PeakRate and PeakEnv with a per-class detection accuracy close to 68% (26 out of 38 bins, *p* < 0.05, permutation test). The per-class detection accuracy is the probability of correctly identifying the time points of a particular class of landmarks (e.g., all envelope peaks) in our 20 ms time windows (see Materials and Methods).

**Figure 3.**
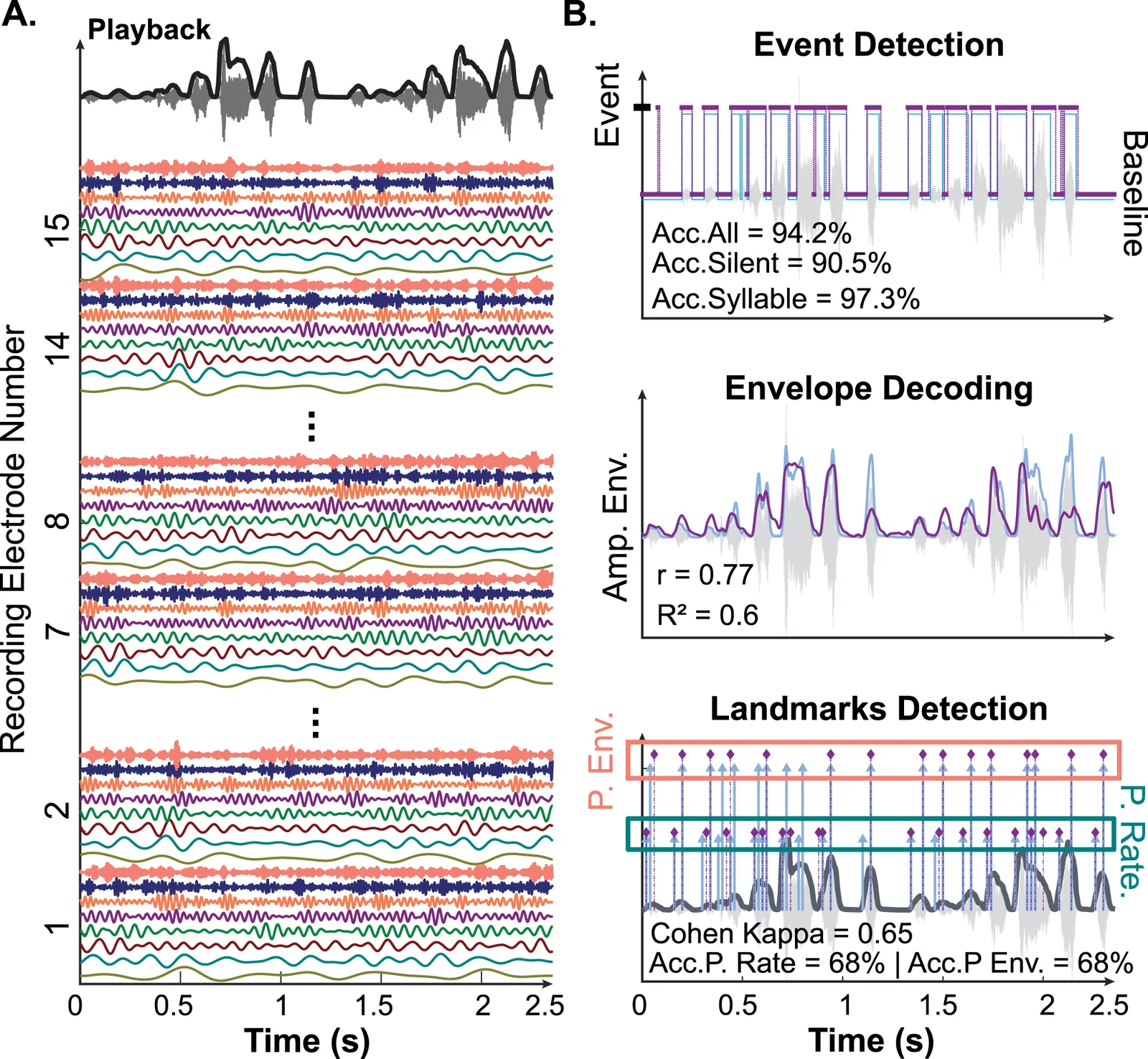
***A***, Song amplitude waveform and envelope for an example song stimulus (top graph: Playback) and the corresponding LFP of six electrodes with LFP decomposition into eight frequency bands. The LFP signals decomposed into these frequency channels are the input features of the decoder. ***B***, An example of the results for decoding three temporal features of song stimuli from LFP neural signals (blue, actual feature; purple, decoded feature). The performances of the decoders are shown using relevant measures of performance for each feature in blue text. For the song Syllable Event Detection (top row), the decoded feature consists of two states (Syllable and Silence) sampled every 10 ms. For the song envelope decoding (middle row), the decoded feature is the amplitude value of the amplitude envelope. On the landmark detection plot (bottom row), two envelope landmarks, PeakRate and PeakEnv, sampled at 20 ms, were decoded. Acc stands for accuracy, and it is the probability of correctly detecting events or landmarks. In the Landmarks Detection, the Cohen's kappa is a measure of average per-class accuracy that takes into account chance levels. Here the three mutually exclusive classes are PeakRate, PeakEnv, and all other points in time.

Across all birds and all sites, we recorded neural responses for 3,660 trials and obtained decoding performance metrics as in Figure 3*B* for each trial and LFPs and MUAe separately (Fig. 4). In Figure 4*A*, each trial is visually represented as a dot on the scatterplot, while in Figure 4*B* the dots correspond to the average performance at a recording site across all trials obtained at that site for both the LFPs and MUAe. The decoding performance based on LFP and MUAe is rather similar (see *r* and effect size values in Fig. 4*A*). MUAe-based decoding slightly outperforms LFP decoding for reconstructing the envelope (mixed-effect modeling with recording session as random effect: $\chi^{2}(1)=5.23,\;p=0.022;\Delta r=0.09\;\pm 0.022\;SE$). No significant difference was observed between LFP and MUAe in decoding both time-locked features, event detection, and envelope landmark detection (mixed-effect modeling with recording session as a random effect; event detection, $\chi^{2}(1)=0.25,\;p=0.62$; landmark detection, PeakRate $\chi^{2}(1)=0.08,\;p=0.77$; PeakEnv, $\chi^{2}(1)=0.10,\;p=0.75$).

**Figure 4.**
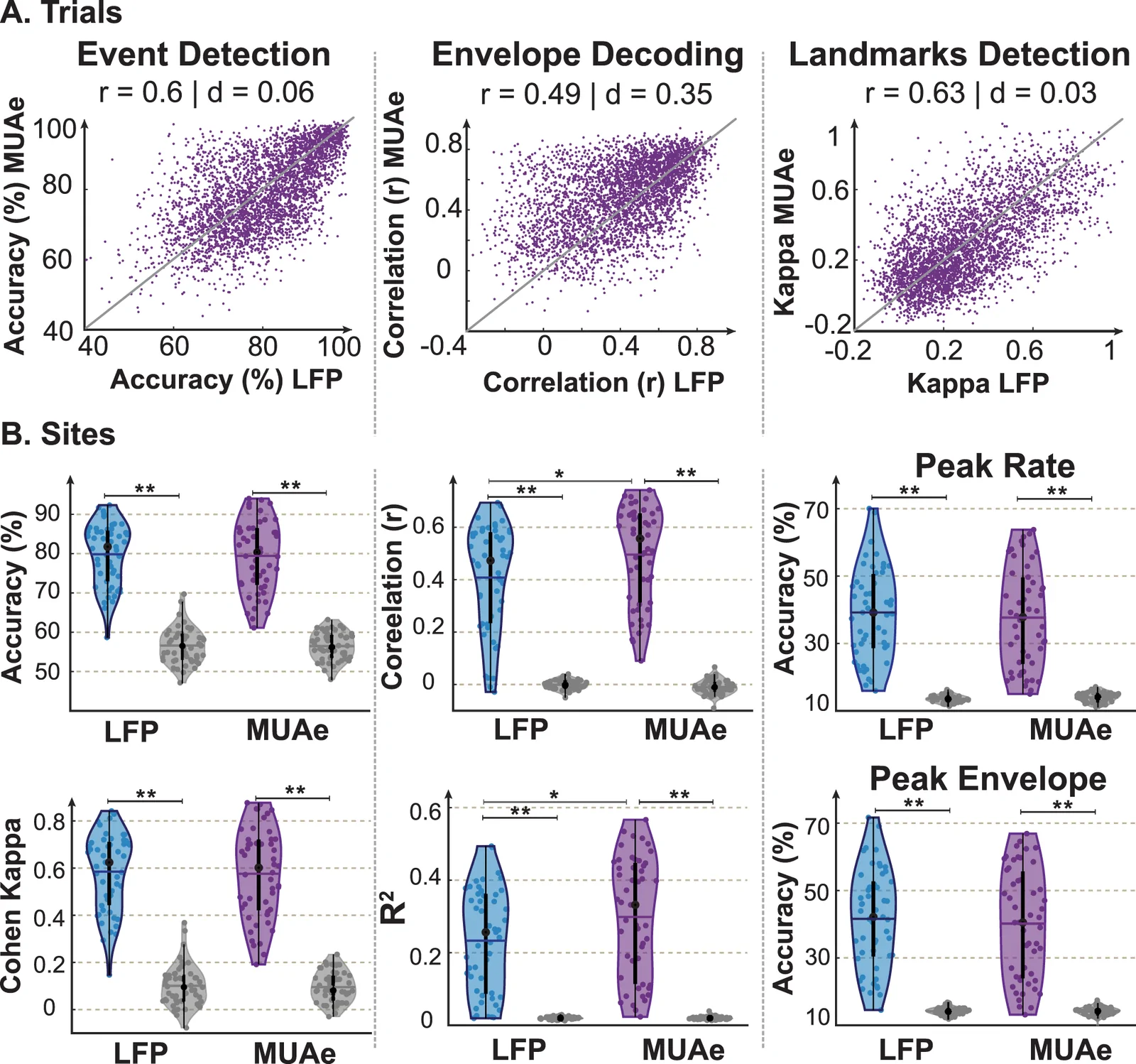
Comparison of LFP with MUAe performance for decoding song temporal features. ***A***, The performance is highly correlated as quantified by the Pearson’s correlation coefficient (*r*) and effect size (*d*). ***B***, Comparison of decoding performance of temporal features of a song by LFP and MUAe features. The performance of the decoders is shown as violin plots to display the distribution of performance across recording sites. The blue violins represent performance based on LFP signals, while the purple violins correspond to MUAe-based performance. The gray violins indicate chance levels, derived from shuffled input features for both LFP and MUAe data. Note that for song events, the chance level for the accuracy of detection is slightly above 50% because the prior probabilities of sound [55.83 ± 2 (SE)%] versus silence [44.17 ± 2 (SE)%] are uneven. Similarly for PeakRate and PeakEnv, the chance level is below 30% and ∼10% because the prior probabilities are 71.33 ± 0.8 (SE)% for nonpeak regions and 28.67 ± 0.8 (SE)% for PeakRate and PeakEnv. First column: Syllable event detection performance, measured in accuracy and Cohen's kappa. Middle column: Amplitude envelope decoding performance quantified by the correlation coefficient (*r*) and the coefficient of determination (*R*^2^). Last column: Envelope Landmark detection accuracy, first row, PeakRate; second row, PeakEnv. *p < 0.05, **p <  1 × 10^−5^ (linear mixed model).

The average performance of song event detection across recoding sites based on LFP and MUAe features was ∼80 ± 1.1% (mean ± SE across trials) and 79 ± 1.3% (mean ± SE) accuracy, respectively, and significantly different compared with shuffled inputs (Fig. 4*B*, left column). The average performance of the amplitude envelope decoding across recording sites based on LFP and MUAe features were significant as compared with shuffled input data (Fig. 4*B*, middle column). In relation, the mean *R*^2^ values between the actual and predicted amplitude envelope based on LFP and MUAe features are ∼0.23 ± 0.02 (mean ± SE) and 0.3 ± 0.02 (mean ± SE), respectively (Fig. 4*B*). Both envelope landmarks were detected with high accuracy (Fig. 4*B*, right column). The PeakEnv and PeakRate detected based on LFP features exhibit an accuracy of ∼42 ± 2% (SE) and 39 ± 2% (SE), respectively (chance level 10% assuming one knows the number of events but not their time). Similarly, for MUAe features, the PeakEnv and PeakRate detection accuracy are ∼40 ± 2% (SE) and 38 ± 2% (SE), respectively. The decoder accuracy is slightly greater for PeakEnv than PeakRate with a difference of ∼2.5%, and both LFP and MUAe as features (mixed-effect modeling with Bird as random factor: LFP, $\chi^{2}(1)=12.116,\;p=0.0005$; MUAe, $\chi^{2}(1)=15.817,\;p<10^{-4}$). We verified that the nonlinear operations performed by the BiLSTM were useful for extracting temporal features: its decoding performance was much better than the one obtained from a linear response model (Fig. S1). In addition, the decoding performance was stable throughout the recording time (Fig. S2), and some song stimuli were systematically better segmented than others (Fig. S3).

### Functional anatomy of temporal decoding

The observed variability in decoding performance across recording sites (Fig. 4*B*) incorporates the variance expected across birds, neural noise, and, potentially, systematic differences across recording sites as we sampled a large region of the avian auditory pallium. Temporal feature decoding using both LFP and MUAe consistently demonstrated stronger contributions from electrodes found in the central region of the electrode array and at intermediate depths. For song event detection, LFP signals from medial sites [0.5 mm mediolateral (ML), 0.75–1.5 mm caudorostral (CR), 0–1.5 mm dorsoventral (DV)] exhibited peak performance at depths of 0.75–1.5 mm. High-performance MUAe decoding similarly implicated central channels (0.5–1.5 mm CR) with broader spatial distribution across ML (0.5–1 mm) and DV (0.75–2 mm) axes. Regions exhibiting significant sensitivity to PeakRate and PeakEnv overlapped substantially at mid-depths (0.75–1.25 mm CR), while MUAe-based performance showed less focal spatial patterns (Fig. 5).

**Figure 5.**
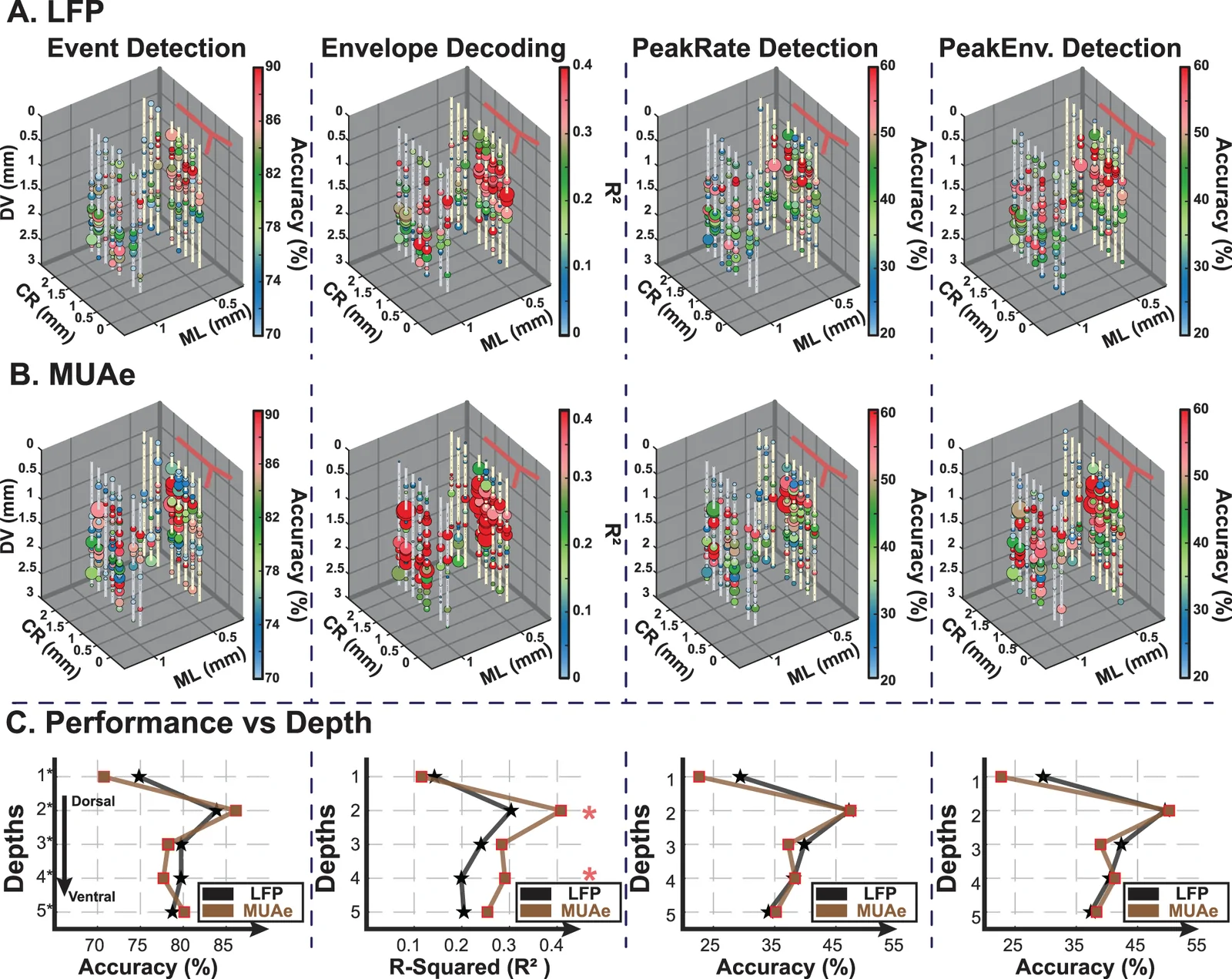
Decoding performance of temporal song features across brain recording sites using LFP (***A***) and MUAe (***B***) signals. Each dot denotes a recording channel, scaled by its contribution to the decoding performance and color-coded by performance for the entire site. The contribution of a single electrode is assessed by the drop in decoding performance following the permutation of that electrode features (see Materials and Methods, Recording electrode contribution). (***C***) Comparison of decoding performance as a function of the depth of the electrode array. Asterisks (*) denote significant differences in decoding performance between LFP-based and MUAe-based decoding at various depths (mixed-effect modeling *p* < 0.05). Full 3D is shown in supplementary videos.

Figure 5*C* stratifies decoding performance by DV depth of the electrode positions subdivided as Depth 1 (0.125–0.5 mm), Depth 2 (0.625–1 mm), Depth 3 (1.125–1.5 mm), Depth 4 (1.75–2 mm), and Depth 5 (>2.25 mm). For all decoded temporal features and both LFP and MUAe as input signals, decoding performance is superior in Depth 2 compared with other depths (mixed-effect modeling with depth and sex as fixed effects, bird as random effect, and decoding performance as response: depth effect *p* < 0.05, sex effect *p* > 0.076, post hoc analyses revealed significant contrasts at specific depths).

To quantify coding fidelity at the level of individual electrodes and later specific regions of the auditory pallium, we computed an EDI measuring the electrodes contribution as well as the overall decoding performance (see Materials and Methods and Fig. 5*A*,*B*). EDI was computed for all 671 auditory-area electrodes across 50 sites and birds for each of the eight signal conditions (Fig. 5; {LFP, MUAe} × {Event, Envelope, PeakEnv, PeakRate}). Recording sites were assigned to Field L (defined here as sub areas L2a,L2b, L3 combined; *n* = 222), L1 (*n* = 126), NCM (*n* = 226), or CMM (*n* = 97) based on histologically verified probe trajectories and estimated depth of recording sites (see Materials and Methods; Fig. 1, Table S1).

The brain region was a significant predictor of EDI for all four temporal features and both LFP and MUA in mixed-effect linear models with sex and recording depth as covariates and session and bird as nested random effects (eight omnibus tests all *p* < 0.001; see Materials and Methods). The largest effects were observed for MUAe-derived Envelope decoding (*F*_(3,643.0)_ = 13.73, *p* < 0.001, marginal *R*^2^ = 0.069) and LFP-derived Envelope decoding (*F*_(3,640.7)_ = 12.21, *p* < 0.001, marginal *R*^2^ = 0.100). Field L (L2a,L2b,L3) consistently exhibited the highest EDI. For the LFP Envelope decoding, the EMMs were as follows: Field L = 0.075 [95% CI: 0.056, 0.094], L1/NI = 0.046 [0.025, 0.067], NCM = 0.052 [0.033, 0.072], and CMM = 0.020 [−0.002, 0.042]. An analogous gradient emerged for MUAe Event decoding, 21.9% [17.5, 26.4] for Field L, 4% [10.3, 20.5] for L1/NI, 16.8% [12.4, 21.3] for NCM, and 7.0% [1.5, 12.6] for CMM. See additional EMM values and tests in Table 1.

**Table 1.** EMMs by brain region and primary versus secondary contrast (dedicated two-group)

		L, L2a, L2b, L3 (primary)		L1 + NI (primary)		NCM (secondary)		CMM (secondary)		Primary EMM	Secondary EMM	LMM F(1, df)/*p*	Cohen’s *d* [95% CI]
Modality	Feature	EMM	SE	EMM	SE	EMM	SE	EMM	SE	EMM	EMM	*F*/*p*	*d* [lo, hi]
LFP	Event	25.81	2.44	20.15	2.82	21.58	2.43	13.64	3.04	23.82	19.18	***F*_(1, 665.7)_** **=** **7.38, *p*** **=** 0.007**	0.220 [0.023, 0.417]
LFP	Envelope	0.075	0.01	0.046	0.01	0.052	0.01	0.02	0.01	0.065	0.043	***F*_(1, 655.7)_** **=** **13.03, *p*** **<** 0.001 ***	0.296 [0.094, 0.498]
LFP	PeakEnv	13.13	1.12	10.37	1.39	11.21	1.11	6.4	1.54	12.21	9.86	***F*_(1, 654.1)_** **=** **5.65, *p*** **=** 0.018*	0.195 [−0.006, 0.396]
LFP	PeakRate	13.03	1.42	11.23	1.62	11.22	1.41	5.32	1.75	12.37	9.53	***F*_(1, 655.0)_** **=** **8.79, *p*** **=** 0.003**	0.244 [0.040, 0.447]
MUAe	Event	21.94	2.01	15.4	2.48	16.85	2	7.02	2.75	19.68	13.97	***F*_(1, 659.8)_** **=** **10.57, *p*** **=** 0.001**	0.261 [0.072, 0.451]
MUAe	Envelope	0.082	0.01	0.038	0.01	0.043	0.01	0.012	0.01	0.067	0.033	***F*_(1, 657.6)_** **=** **17.66, *p*** **<** 0.001***	0.338 [0.149, 0.528]
MUAe	PeakEnv	12.85	1.11	8.71	1.4	8.91	1.11	5.43	1.57	11.43	7.86	***F*_(1, 657.6)_** **=** **12.21, *p*** **<** 0.001***	0.281 [0.092, 0.471]
MUAe	PeakRate	11.89	0.99	8.24	1.24	8.33	0.97	4.65	1.4	10.6	7.19	***F*_(1, 651.8)_** **=** **13.64, *p*** **<** 0.001***	0.296 [0.110, 0.482]

EMMs and SEs are in original EDI units: envelope conditions (LFP and MUAe) report raw decimal values; all other conditions report percentage-scale units. EMMs are adjusted for sex and recording depth at their grand means. Primary, field L + NI pooled; secondary, NCM + CMM pooled. Dedicated two-group LMM uses the same fixed-effect and random-effect structure as the four-region model. Cohen's *d* with bootstrapped 95% CI. Significance codes: ***p < 0.001; **p < 0.01; *p < 0.05; ns, not significant.

The primary (Field L including L1) versus secondary auditory region (NCM and CMM) planned contrast was significant for all four temporal features and the two neural signal modalities (all *p* ≤ 0.007; Fig. 6). For the MUAe Envelope condition, a two-group LMM demonstrated that primary-region recording sites carried nearly twice the EDI of secondary-region recording sites (primary EMM = 0.067, Secondary EMM = 0.033 EDI units; *F*_(1,657.6)_ = 17.66, *p* < 0.001; Cohen's *d* = 0.338, 95% CI: 0.149, 0.528) and for MUAe PeakEnv (*F*_(1,657.6)_ = 12.21, *p* < 0.001; *d* = 0.281, 95% CI: 0.092, 0.471). We did not find any significant main effects for sex (*p* > 0.120). Additional statistical tests for LFPs and for comparing differences across subregions are shown in Table S2.

**Figure 6.**
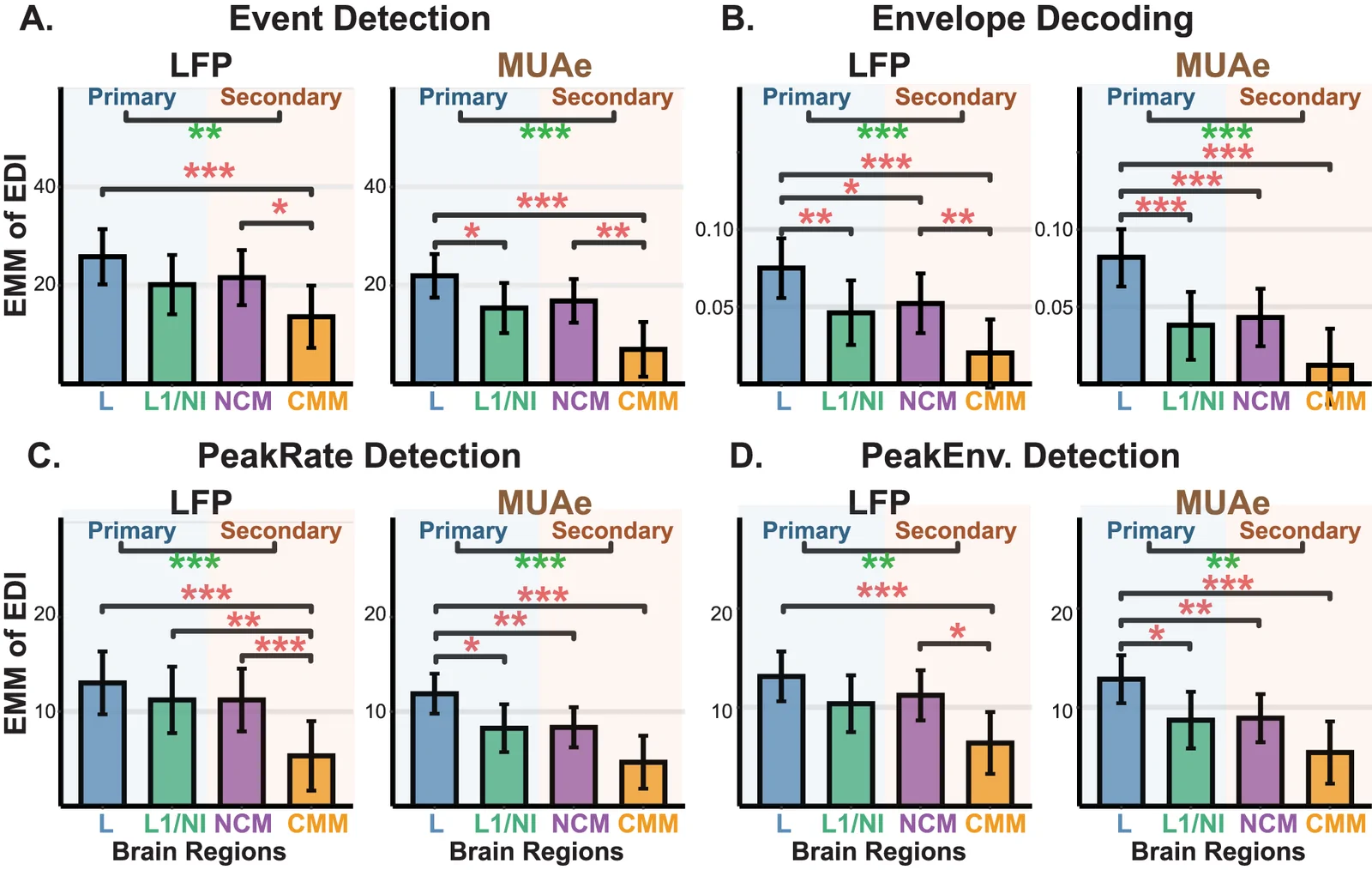
EMMs of the EDI across auditory brain regions for the four temporal features (panels ***A–D***) and the two neural signals: LFPs and MUAe. Each panel shows the EMMs as filled color bars and 95% confidence intervals as error bars both derived from linear mixed models with brain region (four levels), sex, and recording depth as fixed effects and nested random intercepts for bird and recording session. Significance annotations indicate the overall brain region Type III *F* test result (*p < 0.05, **p < 0.01, ***p < 0.001; see also Table 1, Table S2).

### Response properties of neural signals in high-decoding recording sites

Finally, we examined the relationship between multichannel MUAe decoding performance and the response properties of single units. We first related the event detection and envelope decoding performance to the information rate of individual units (Fig. 7). Specifically, we calculated the MI between the song events or amplitude envelope and the spiking activity of single units (see Materials and Methods). There is a strong correlation between single-unit information rates and decoding performance, as assessed by Pearson’s *r* (statistical values are provided in Fig. 7*B*). We also analyzed the temporal relationship between spiking activity and the PeakEnv and PeakRate using cross-correlation. Similarly, we observed a strong correlation between the degree of single-unit locking to these envelope features and the decoding performance (Fig. 7*A*, PeakRate and PeakEnv Detection). Next, we examined the anatomical locations of single units that exhibited high MI or showed highly correlated activity with the stimulus's temporal features (Fig. 7*B*): units tracking envelope landmarks are concentrated in specific areas within the auditory forebrain of zebra finches, particularly at depths ranging from 0.7 to 1.3 mm in middle sites, which aligns with the central region of Field L. To further examine that spatial relationship, we estimated the latency and modulation depth of landmark locked responses for all single units assigned to distinct regions of Field L, NCM, and CM. As shown in Figure S5, the latency increased and modulation depth decreased as one moved from the central region of Field L (putative L2) to secondary regions in the auditory nidopallium and mesopallium. This localization suggests region-specific encoding strategies within the auditory processing network of zebra finches. Single units at recording sites with high information rates exhibit strong phase locking to the amplitude envelope of the song stimulus and precisely respond to song events (Fig. 7*C*, first and second column). Furthermore, the spiking activity closely aligns with PeakEnv times and peaks ∼10 ms after PeakRate events (Fig. 7*C*, third and fourth column). This consistent delay across neurons generates a synchronized population response that facilitates accurate temporal encoding of song features in the neuronal response from groups of neurons.

**Figure 7.**
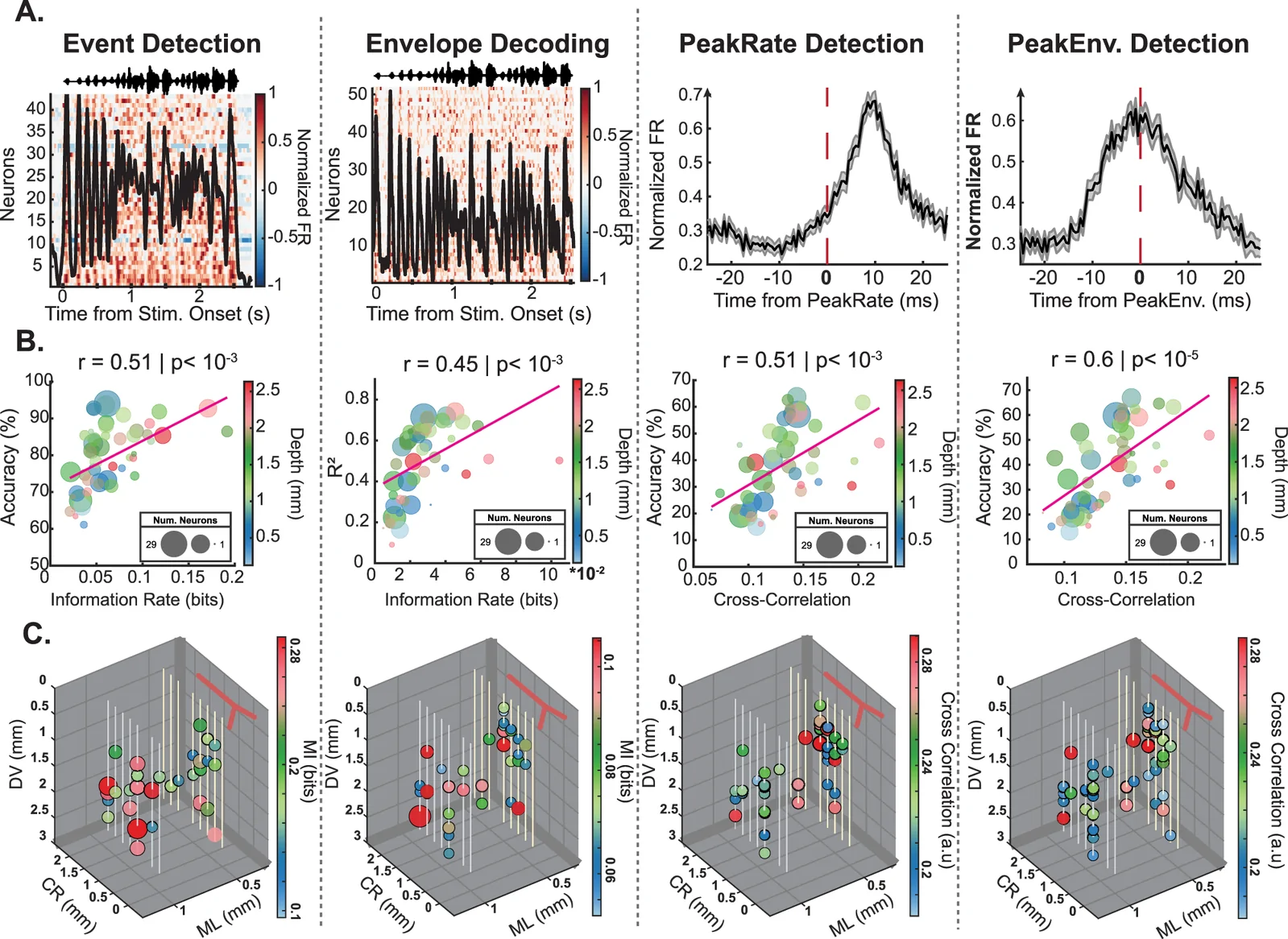
Single-unit activity encoding capabilities for temporal song features and their relation to decoding performance by MUAe. ***A*1-2**, Normalized firing rates (FR) of a subset of neurons that track song events and envelopes. Each row in the matrix corresponds to a single neuron, and the solid line represents the average FR for all *n* = 43 neurons. ***A*3-4**, Cross-correlation between envelope features and spike activity of single-unit that tracks each envelope landmark. Single-unit spike activity during song processing aligns with envelope landmarks; the *x*-axis shows time from envelope landmarks, and the *y*-axis shows the normalized FR. ***B***, Comparison of MUAe decoding performance with single-unit information rates for encoding temporal features of the song across recording sites, with bubble size indicating the number of single units and color representing recording depths. ***C***, Spatial maps showing neurons that encode temporal features of songs with high information rates or correlations.

Since single units with high information rates for temporal song features are located at recording sites that also show high multichannel population decoding performance, we investigated whether the MUAe and LFP signals also showed correlations with decoding performance. We categorized recording sites by their decoding accuracy using the MUAe (high, mid, low). These sites also showed greater LFP ITPCz, indicating more reliable time-varying neural responses, especially in the alpha (8–13 Hz) and beta (13–30 Hz) bands (Fig. S6).

Furthermore, analysis of zebra finch song structure revealed that temporal information in the introductory notes and the first motif was decoded more accurately than the second motif (Fig. S7). Neural representation of temporal information is therefore not time-invariant but might depend on context. In this case, neural synchronization for segmentation and consequently identification might be more important for the initial section of the song stimuli, when the receiver has less information on the identity or quality of the sender (Yu et al., 2020).

### Computational steps for decoding temporal features

Our findings demonstrate that it is possible to use DNNs to decode behaviorally relevant temporal features of song stimuli from multichannel LFP or MUAe activity. We also found correlations between the accuracy of decoding and the properties of neural responses. To gain insight into the computational processes underlying decoding, we tested whether DNN layer operations could be linked to understandable neural response transformations.

To visualize the changes in representation performed by the stacked BiLSTM architecture, we applied PCA first to the input LFP features and then to the hidden neuron activities in each BiLSTM layer. We show an “average” response corresponding to the first dimension of this PC in Figure 8*A*. This dimensionality reduction of the DNN activity allowed us to visually assess how the temporal patterns of the song stimuli are captured by each layer. Next, we examine the activity of all hidden neurons, focusing on their degree of synchrony. For this purpose, we applied *t*-SNE to the time series of each neuron, both for visualization and clustering. As shown in Figure 8*B*, the activity of first-layer hidden neurons (first row) contains many clusters reflecting low synchrony and thus low redundancy in their activity. However, the temporal information is present in those signals, as made evident by the emergence of clusters of synchronized activity in the deeper layers (Fig. 8*B*3). Moreover, peaks (red) and troughs (blue) in the activity profiles associate with temporal features of the amplitude envelope, such as onset, offsets, and amplitude (Fig. 8*B*3).

**Figure 8.**
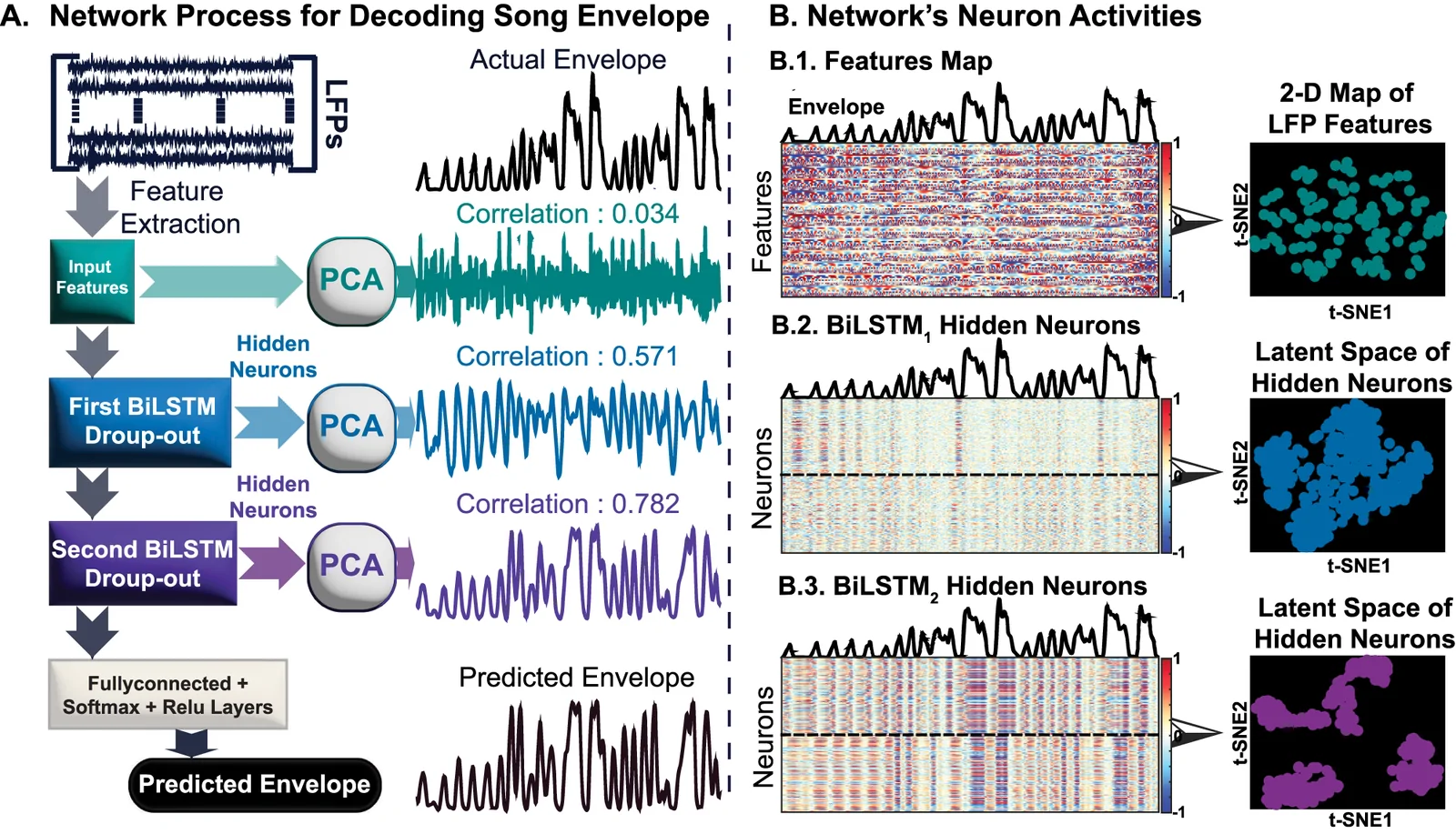
Illustration of the processing occurring at different layers of the DNN. ***A***, To reduce dimensionality, PCA is utilized to downsample the input features and the network's hidden neurons' activity matrix to a single dimension (time scale). The correlation coefficients between the actual envelope and the hidden neurons are depicted at the top of each plot. ***B***, This illustration shows the transformation from features to hidden neuron activities of the network for one song and one trial. ***B*.1**, The first column presents the input LFP features in a color matrix. The LFP features from distinct frequency bands and recording channels are shown in distinct rows. The envelope of the song is decoded on top of the matrix with a solid black line. The 2-D maps use *t*-SNE to show each time series as a single point. ***B*.2**, Same as ***B***.**1** for the activities of the artificial neurons in the BiLSTM layer. ***B*.3**, Same as ***B***.**2** for the second BiLSTM layer, revealing fiber clusters of highly synchronized artificial neuronal responses.

Next, we compared the responses of artificial neurons in the second BiLSTM layer to the responses of actual single units. For this comparison, we used the envelope decoder trained on the multichannel MUAe recorded in a single session (Session 46) rather than the LFP-based decoder illustrated in Figure 8. The actual single units included in the comparison were pooled across all birds and recording sessions. Because MUAe and single units are both derived from the 500–9,000 Hz neural signal, a small fraction of single units recorded in Session 46 (29 of 423, 6.9%) contributed indirectly to the decoder input as part of the multiunit signal. All other single units were recorded in different sessions and did not contribute to the decoder input. Our goal here was not to obtain a one-to-one match between all auditory neurons found in the avian pallium and the hidden units of our decoder but instead assess whether a subset (or multiple subsets) of actual auditory neurons have response properties that match artificial neurons found at the output of the decoder. Those actual neurons might thus reflect the output of biological computations optimized to extract temporal information, similarly to the artificial units of the BiLSTM that were optimized to predict the timing of acoustic signal landmarks. These single neurons would be sensitive to synchronous activity of a larger group of neurons that can be captured here in the MUAe. To assess this similarity, we applied the same nonlinear embedding (*t*-SNE) used in Figure 8*B* jointly to the time series of the artificial and actual neural responses. We found some overlap between the artificial and real neurons (Fig. 9*A*). To further determine whether the artificial and actual neurons belonged to similar groups, we applied unsupervised clustering (DBSCAN) to the joint data. Since this clustering was based on *t*-SNE embeddings of temporal response profiles, it will identify groups of neurons with similar average temporal response profiles to the same stimuli; note, however, that this analysis characterizes response pattern similarity and does not constitute a statistical characterization of pairwise coactivation or functional connectivity based on single-trial data that would be required to describe neuronal assemblies. The unsupervised clustering revealed three mixed clusters containing both artificial and real neurons in similar proportions (Fig. 9*B*). The activities of neurons from these three mixed clusters were highly phase-locked to the amplitude envelope, and the activities of these artificial and real neurons were highly correlated (Fig. 9*C*; *r* = 0.49, 0.53, 0.63 for Clusters 1, 2, and 3, respectively). The mixed clusters did not depend on units that contributed to the decoder input. After excluding the 29 single units recorded in Session 46 (6.9%), mixed clusters emerged in all 10 *t*-SNE initializations (2-3 per run; median *r* = 0.50, range from 0.24 to 0.70, across 26 mixed clusters), and Session 46 units were not overrepresented in any mixed cluster of the full data (one-sided Fisher's exact test, all *p* > 0.05). This analysis identifies a subset of auditory neurons, recorded across multiple sites (Fig. 9*D*), whose stimulus-locked responses match temporal profiles that emerge in a decoder optimized to reconstruct the song envelope. This correspondence does not demonstrate that the auditory pallium implements the same computations as the BiLSTM. It is, however, consistent with the hypothesis that some pallial neurons integrate synchronous activity across the local population to represent envelope landmarks, a hypothesis that will require direct testing.

**Figure 9.**
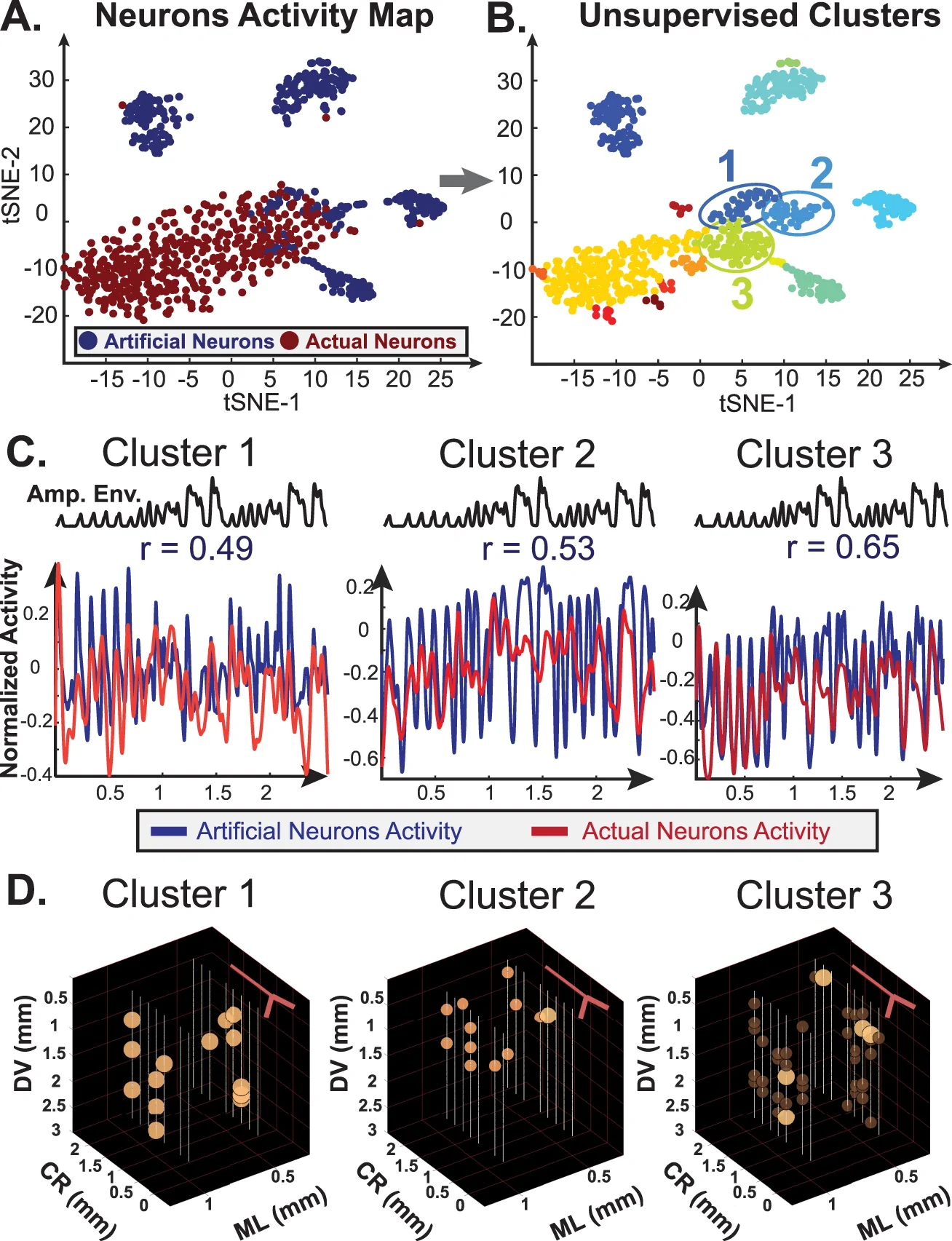
Comparison of artificial and actual neuron activity. ***A***, Latent space representations of actual and artificial neurons visualized using a *t*-SNE. Red dots represent actual single units pooled across all birds and recording sessions, while blue dots denote artificial neurons from the second BiLSTM layer of the envelope decoder trained on multichannel MUAe from a single recording session. ***B***, Unsupervised clustering of both actual and artificial neurons using the density-based clustering nonparametric algorithm (DBSCAN). Circles indicate mixed clusters containing both actual and artificial neurons. ***C***, Actual and artificial neurons' average normalized activity patterns within the mixed clusters. The blue line represents artificial neuron activity, and the red line shows actual neuron activity. The amplitude envelope of the song is displayed at the top of the panel. ***D***, Brain maps showing the locations of neurons of the mixed Clusters 1-3. Lighter colors indicate a higher density of neurons at the recording sites.

## Discussion

Using a decoding approach, we have first shown that the amplitude envelopes of a communication signal, the male zebra finch song, can be decoded with a high level of accuracy from single-trial neural signals recorded from the primary and secondary avian auditory pallium. The song amplitude envelopes could be decoded with an average cross-validated *R*^2^ across all recording sites of ∼0.3, with the best sites reaching ∼0.6. Since auditory neurons become sensitive to specific amplitude modulations as a result of multiple computational steps that are implemented in the ascending auditory system (reviewed in Joris et al., 2004), the ability to decode the amplitude envelope for cortical auditory responses is expected. However, there are very few animal studies that have directly attempted to quantify the quality of the reconstructed amplitude envelope of a naturally occurring and behaviorally relevant sound. Given the relatively low number of neural signals (compared with the number of avian auditory pallial neurons) used in our decoder, this reconstruction performance is impressive. Comparable performance in stimulus reconstruction was found in the mammalian auditory system, with recordings from 250 neurons yielding reconstruction up to *r* = 0.8 (*R*^2^ ∼0.64; Mesgarani et al., 2009).

The decoding of the amplitude envelope of vocal signals has been examined in more detail in human research (Pasley et al., 2012; Anumanchipalli et al., 2019) where the entrainment of cortical activity has been correlated with speech intelligibility both in normal and impaired hearing subjects (Verschueren et al., 2019; MacIntyre et al., 2024). Such findings support the idea that areas of the human auditory cortex might be specialized for speech processing and comprehension (Hamilton et al., 2021). Similar performances for human speech envelope decoding have been achieved from recordings in the superior temporal gyrus of nonhuman primates (Heelan et al., 2019). Here we show that auditory neurons in the avian auditory pallium are, in a quantifiable manner, just as entrained by the envelope of the sound of one of their communication signals. Thus, we suggest that the high-fidelity decoding of the amplitude envelope of behaviorally relevant or salient sounds is not a unique feature of human cortical regions specialized for speech but a property of auditory cortical responses in vertebrate species that rely heavily on vocal communication calls.

### Neural representation of sound amplitude landmarks for segmentation

The neural representation of the sound amplitude envelope can provide key information for the identification of acoustic signals in both humans and animals. In humans, preserving the temporal shape of the speech envelope while distorting much of the spectral information results in satisfactory speech comprehension (Shannon et al., 1995; Elliott and Theunissen, 2009). Likewise, in animals, one can use the neural decoded amplitude envelopes to recover the stimulus identity (Penikis and Sanes, 2023). However, it is also evident that the identification of auditory objects in human speech is based not only on their envelope but also its comprehensive spectral and temporal structure (Elliott and Theunissen, 2009). Could there be another functional role of the strong neural entrainment observed in auditory cortical areas for communication sounds? In humans, it was discovered that electrocorticography responses in the superior temporal gyrus could be well predicted by convolving impulse responses with event times occurring at acoustic edges (Oganian and Chang, 2019). Analyses of responses to slowed-down speech further showed that neural synchrony observed in the human auditory cortex was best explained by neural responses phase locking to these acoustic edges rather than by an oscillatory entrainment to the envelope (Oganian et al., 2023). Thus, it is postulated that the synchronous activity that is observed across many auditory cortical neurons reflects the representation of edges more so than the actual time-varying envelope. This acoustic edge representation could play a key role in segmenting a sound stream into units that can be analyzed for their spectral–temporal context and ultimately identified as a particular auditory object. Indeed, in zebra finches, once calls are segmented into units, they can be identified as belonging to a particular call type with a high degree of accuracy (Robotka et al., 2023). For human speech processing, segmenting the sound stream is a key computational step in the sound-to-meaning (i.e., lexical retrieval) transformation (Pisoni and Luce, 1987; Gwilliams et al., 2022).

With this framework in mind, we examined how auditory edges are decoded in the zebra finch. Inspired by Oganian and Chang (2019), we decoded the times of peak values (PeakEnv) and of peak derivatives (PeakRate) in the song amplitude envelope. The time of both the PeakEnv and PeakRate could be decoded with an average accuracy of ∼40%, with best accuracies reaching 70%. In addition, the song could be segmented into segments of sounds and silence with an average accuracy of ∼80% and a peak reaching ∼90% (Fig. 4). Thus, neural responses in the avian auditory pallium can be used to precisely identify sound landmarks (“edges”) and cut the sound stream into segments.

This segmentation/identification model of the auditory cortex needs to be further investigated. For example, in this study, we have only analyzed responses to playbacks. Although these elicited both orienting and vocal responses in our freely moving birds, segmentation will be even more essential during natural exchanges between two or more birds. We have also chosen landmarks that are based solely on the amplitude envelope. It is highly possible that other landmarks events, for example, defined by abrupt changes in spectral structure but with minimal changes in amplitude, will also be critical for identifying behaviorally relevant song elements.

### Mechanisms and comparison between LFPs and MUAe

To be able to encode temporal events, the neural response across neurons (or recording sites) must be synchronized. This synchronization does not necessarily require perfect coincident activity but does require a systematic phase alignment. We analyzed the phase angles of neural signals at specific frequencies, with ITPCs measuring the consistency of these phase angles across trials. High ITPC indicates stable neural alignment with stimuli, supporting tasks that require precise timing (Herrmann et al., 2013; Van Diepen and Mazaheri, 2018; Sjuls et al., 2024). The fact that one can recover an average firing rate response triggered by the event shows that this systematic phase exists to some extent (Fig. 7). Since the BiLSTM can efficiently capture these systematic delays/advances when combining neural responses, it provides a powerful mechanism for decoding envelopes of songs (this study) and speech (Anumanchipalli et al., 2019; Thornton et al., 2022).

We found that the decoding accuracy was very similar using LFP versus MUAe features for the neural signal. A priori, either signal could have yielded better results. On the one hand, the LFP signal at one electrode site integrates neural activity samples from more neurons since its lower frequencies reflect the summed contribution of synaptic input whereas higher frequencies correlate with synchronized spiking activity (Denker et al., 2011). This aggregation could reduce the signal-to-noise ratio if similar phase offsets are found across neurons. On the other hand, the MUAe recorded at the same site reflects the spiking output of a smaller number of neurons but with higher temporal precision. Previous research has demonstrated that increased temporal resolution significantly enhances MUAe decoding performance, with optimal results achieved at resolutions under 10 ms (Smith et al., 2013; Ahmadi et al., 2021). In tasks involving auditory stimuli, MUAe consistently outperforms LFP in both decoding accuracy and responsiveness to temporal features (Burns et al., 2010). In our data, the relatively small number of units recorded simultaneously would give an advantage to the LFP signals, but this advantage appears to balance out the benefit from the increase in temporal precision in MUA.

The fact that a relatively small number of neural signals is sufficient for temporal decoding with high precision suggests that there must be a good fraction of neurons that are tuned to amplitude modulation. In prior work, ∼40% of auditory neurons in the primary auditory pallium of zebra finches were classified as broadband neurons from their spectrotemporal receptive fields (Woolley et al., 2009). These neurons show little frequency selectivity but are sensitive to overall changes in the amplitude of the sound at distinct amplitude modulation frequencies. They are, therefore, excellent candidates for encoding the amplitude envelope of the songs. In addition, to encode events, one could combine responses from neurons with distinct amplitude modulation tuning. These neurons’ tendency to respond at the early phase of the amplitude modulation cycle (Penikis and Sanes, 2023) would result in a synchronized population of neurons to events.

### Anatomical and cell specificity

Although song amplitude envelope and song landmark information could be reliably decoded from neural recordings obtained at various locations in the avian auditory pallium, we did observe that the higher decoding performance was found at electrode depths that yielded the largest sampling from the primary auditory areas, Field L (Figs. 5 and 6). As different regions in both the mammalian cortex (Osman et al., 2018) and avian auditory pallium (Sen et al., 2001; Nagel and Doupe, 2008; Kim and Doupe, 2011) are known to code different time scales in the amplitude modulation domain, the anatomical specialization observed here might simply reflect a particular sampling of time scales that is optimized for capturing the dynamics on the song envelope. Alternatively, we propose that, in primary auditory regions, the “segmentation” fraction of the neural response is more explicitly segregated from the “identification” fraction, whereas segmentation and identification are more integrated in neural responses found in secondary auditory regions CMM and NCM.

At a finer granularity, we found that the single units that had a higher MI rate with the sound envelope had a higher contribution to the decoder performance (Fig. 7). Thus, not only might there be pallial subregions that more explicitly represent the segmentation signal, but within those regions, particular neurons appear to be more specialized for temporal processing. This specialization might also be different for distinct neuron types, such as excitatory versus inhibitory neurons (Spool et al., 2021). In this study, we did not attempt to identify putative candidates for distinct neural classes based on their spike shape (Schneider and Woolley, 2013). Instead, we combined all neuron types recorded simultaneously for our multichannel decoding analyses. In future research, we plan on characterizing the role of specific single neuron types and circuits. We postulate that the auditory pallium in birds and the auditory cortex in mammals are composed of microcircuitry whose function is to implement the segmentation and identification computational steps. Although these two functions are multiplexed in most, if not all, neural signals, the variability in the degree to which segmentations are explicitly represented provides a means to understand the neurobiological implementation of the intertwined segmentation and identification computations.

## Data Availability

All primary electrophysiological recordings (RHD format), the corresponding playback stimuli, and backpack-microphone tracks have been deposited in the Edmond repository under https://doi.org/10.17617/3.UINR2V. Playback metadata are included in the same record as comma-separated values (.csv) files. The source data supporting every figure, the complete decoding outputs, and the single-unit response matrices for all playback stimuli are available in Edmond under https://doi.org/10.17617/3.AUMDAU. All analysis scripts and figure reproduction code are openly available on GitHub at https://github.com/amirmasoud92/ZF_Neural_Decoding.
